# Transformation of Psoralen and Isopsoralen by Human Intestinal Microbial In Vitro, and the Biological Activities of Its Metabolites

**DOI:** 10.3390/molecules24224080

**Published:** 2019-11-12

**Authors:** Lu Liu, Lei Zhang, Ze-Xu Cui, Xiao-Yan Liu, Wei Xu, Xiu-Wei Yang

**Affiliations:** State Key Laboratory of Natural and Biomimetic Drugs, Department of Natural Medicines, School of Pharmaceutical Sciences, Peking University Health Science Center, Peking University, No. 38, Xueyuan Road, Haidian District, Beijing 100191, China; a1534064875@163.com (L.L.); zhangyutian0619@163.com (L.Z.); yanzi_89@163.com (X.-Y.L.); high-xu@163.com (W.X.)

**Keywords:** *Psoralea corylifolia* L., psoralen, isopsoralen, LC-MS/MS, human intestinal flora, oxidative stress

## Abstract

Psoralen (P) and isopsoralen (IP) are the main active ingredients in the dried fruit of *Psoralen corylifolia* L. (PC), with a wide range of pharmacology activities. The intestinal bacteria biotransformation plays a central role in the metabolism of the complex ingredients in traditional Chinese medicine (TCM). Our study aimed to investigated the metabolic profile of P and IP in the intestinal condition, co-cultured with human fecal bacteria anaerobically. Four bio-transforming products were obtained, including 6,7-furano-hydrocoumaric acid (P-1) and 6,7-furano-hydro- coumaric acid methyl ester (P-2), which transformed from P, and 5,6-furano-hydrocoumaric acid (IP-1) and 5,6-furano-hydrocoumaric acid methyl ester (IP-2), which were transformed from IP. It is worth mentioning that IP-2 is a new compound that has not been published. Their structures were analyzed based on their spectroscopic data. Moreover, a highly sensitive ultra-performance liquid chromatography tandem mass spectrometry (UPLC-MS/MS) method was used to characterize the metabolic pathways of P, IP, and their bio-transforming products in the reaction samples. In addition, the dampening effects against the oxidative stress of P, IP, and their bio-transforming products by human intestinal flora were estimated in vitro via the human colorectal cells (HCT116) and heterogeneous human epithelial colorectal adenocarcinoma cells (Caco-2) cell lines. The results showed that the metabolites have stronger activity than P and IP, which possibly provides a basis for elucidating the treating mechanisms of PC extract against inflammatory bowel disease.

## 1. Introduction

Traditional Chinese medicine (TCM) have been used to guide clinical treatment by herbalist doctors for thousands of years. *Psoralea corylifolia* L. (PC), which is part of the family Fabaceae, is extensively used in China, India, and many other countries [1]. In China, the extract of PC fruits is one of the most commonly used tonic herbs, having the effects of kidney impotence and warming spleen, which could treat diarrhea effectively, and has been included by Pharmacopoeia of People′s Republic of China (2015) [2]. Being familiar with its vernacular names in India, the most widely used types are Babachi (Hindi), Bemchi, Bawchi, and so on. [3]. However, several reports have indicated that PC is regarded as an endangered medicinal plant in India, so the research of this plant has attracted much attentions [4,5]. Modern pharmacological studies have shown that the dried fruit of the plant has anti-bacterial [6], anti-diabetic [7], anti-depressant [8], anti-oxidative [9], anti-fungal [10], anti-inflammatory [11], and many other effective activities.

**Figure 1:**
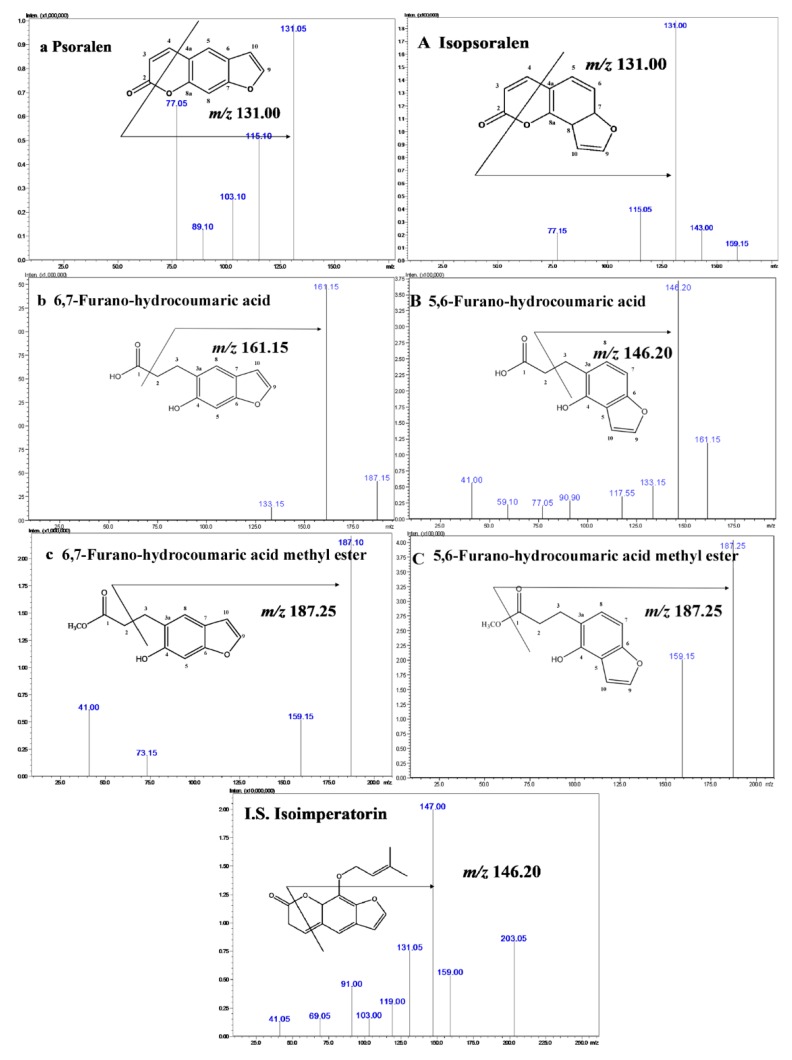
Chemical structures and MS/MS fragmentation patterns of psoralen (P), isopsoralen (IP), and their transformation products.

Phytochemical examinations revealed that various components, including coumarins, flavonoids, phenols, monoterpenoids, quinines, sesquiterpenoids, triterpenoids, aliphatic acid, and some other components, have been isolated from PC [12]. Among them, coumarins have been proven to be the main active constituents of PC, and the highest ingredients in PC are psoralen (P) and isopsoralen (IP; structures shown in Figure 1). Previous studies have reported their therapeutic effects, such as antitumoral, antiamnesic [13,14], antibacterial, and estrogen-like effects [15,16]. More specifically, P and IP present a dose-dependent anticancer activity in oral carcinoma and erythroleukemia cells [17], respectively. Also, P and IP exhibit a significant anti-oomycete activity against *Phytophthora infestans* [18]. P has been demonstrated to adjust the function of osteoblasts in mice that suffer from the tumor, and resulted in the growth inhibition of the breast cancer cell in the bone environment, which could also be understood that P could inhibit the cancer migration of breast cancer to bone [19]. Furthermore, scientists have found that five isolated components from the seeds of PC exhibited a tyrosinase inhibitory activity [20]. The antioxidant activities of seven compounds, including P from PC extracts, were quantitatively estimated by the electron spin resonance (ESR) method, for which the results illustrate that these compounds expressed different degrees of antioxidant activity [21]. Furthermore, it is well established that oxidative stress is common in inflammatory bowel diseases (IBDs), and that antioxidants are usually recommended for the treatment [22], so we have thought more along the line of antioxidant stress. Furthermore, our previous report suggested the potential value of the Fruetus psoraleae compounds in alzheimer′s disease (AD) prevention and treatment [23,24].

The oral administration for drugs is the most convenient method for the majority TCM—their active components are subjected to absorption, distribution, metabolism, and excretion in individuals. Moreover, intestinal bacteria transformation is a crucial link, which lead to generating more active compounds in the alimentary tract. It is a reasonable way to look for new leading compounds based on the physiological disposition process of the compounds [25]. At present, many plasma pharmacokinetic studies of P and IP have been reported [26], and several researches indicate that P and IP show a fast-oral absorption with the enterohepatic circulation. Our previous studies have demonstrated that some coumarins in PC could penetrate the blood–brain barrier rapidly and accumulate in the brain [27]. The tissue distribution essay result shows that the coumarins in the PC extract have a high bioavailability, and they are rapidly and widely distributed into tissues [26,28]. Combined with the recent progresses of pharmacological activities mentioned above, we have every reason to believe that the explication of the dynamic process in the organisms of PC will be a key step to understanding their pharmacodynamic material basis. The transformation of P and IP by intestinal bacteria, however, has rarely been studied. Therefore, it is necessary to investigate the biotransformation of P and IP by incubated human intestinal bacteria in vitro. Moreover, the related activities of P and IP, and their transformation products have been evaluated, such as being able to dampen the effects of oxidative stress.

## 2. Results and Discussion

### 2.1. Metabolic Processing of P and IP by Human Intestinal Bacteria

P and IP were separately incubated with the bacterial suspension anaerobically, and the metabolic samples were incubated for 12, 24, and 48 h, then extracted with ethyl acetate (EtOAc), and analyzed by HPLC. What needs illustration is that several organic solvents were tested, including EtOAc and water-saturated n-BuOH, as well as firstly extracting with EtOAc, then water-saturated *n*-BuOH. Consequently, the bacterial suspension was fully extracted with EtOAc., and water-saturated butanol was not chosen as the extraction reagent, so as to exclude unnecessary impurity. As shown in Figure S1, compared with the control sample (general anaerobic medium (GAM) and IP) and blank sample (incubation solution without analyses), two metabolites from IP were obtained. The metabolic pathway of P was similar to IP. The chemical structures were evident by their spectra of infrared ray (IR), liquid chromatography-mass spectrometry (LC-MS), ^1^hydrogen-nuclear magnetic resonance (^1^H-NMR), ^13^carbon nuclear magnetic resonance spectroscopy (^13^C-NMR), heteronuclear multiple quantum coherence (HMQC), heteronuclear multiple bond correlation (HMBC), and heteronuclear single quantum correlation (HSQC), and compared with the data reported in the published paper, which were consistent with the structure, being 6,7-furano-hydrocoumaric acid (P-1), 6,7-furano-hydrocoumaric acid methyl ester (P-2), and 5,6-furano-hydrocoumaric acid (IP-1). It deserves to be mentioned that IP-2 (5,6-furano-hydrocoumaric acid methyl ester) was confirmed as a new metabolite, which has never been published before. Among them, the amounts of P-1 and IP-1 were relatively large, which were the main biotransformation products of P and IP, respectively. The amounts of P-2 and IP-2 were relatively smaller than that for P-1 and IP-1. Despite this, all of the four transformation products were obtained by our research for probing into the biotransformation mechanism of P and IP in the human intestinal flora, which will be further discussed in the Discussion section. Moreover, the stability of the internal standard (I.S.) in the microflora solution was also performed, but no transformation products were generated. The structures of six compounds and I.S., as well as the ion pairs, are illustrated in Figure 1.

**Figure 2:**
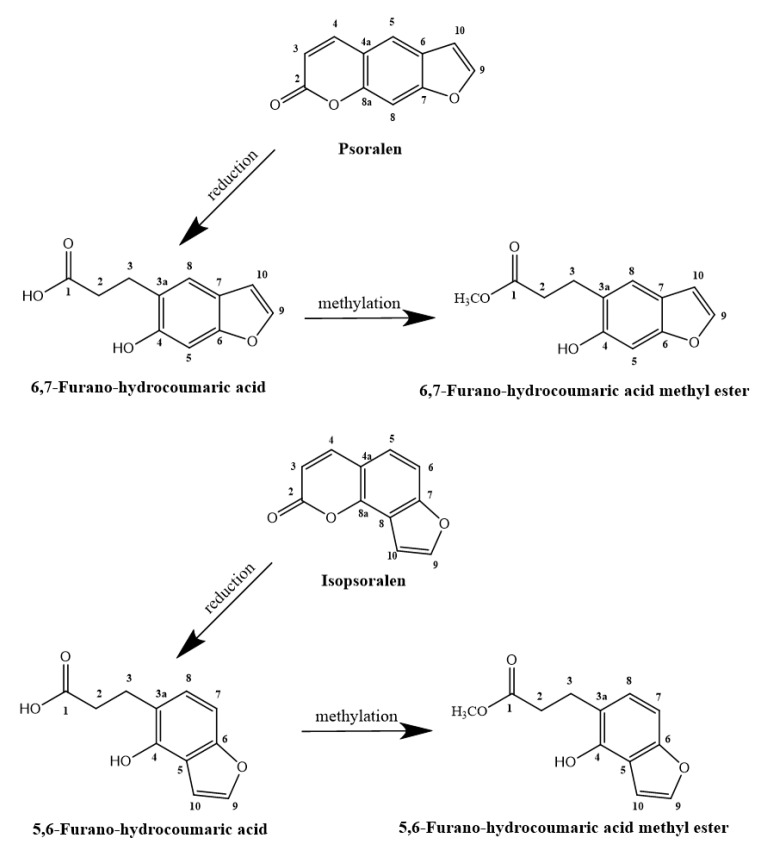
The possible metabolic pathways of P and IP by human intestinal bacteria.

Biotransformation products P-1 (101 mg) and P-2 (15 mg) were isolated from the extract of P, and IP-1 (121 mg) and IP-2 (8 mg) were isolated from the extract of IP. The chemical structures of the transformed products are listed in Figure 2. The transformation processes were monitored by an Agilent HPLC-DAD system (Agilent, Waldbronn, Germany) at 245 nm, the details of which are discussed in the supporting information (Figure S1, Table S2, and the section of “Procedures and Conditions of Chemical Separation”).

**P-1**: C_11_H_10_O_4_, white powder. IR (KBr) *υ*_max_ 3420.82, 1080.81 cm^−1^. ESI MS *m*/*z* 205.15 [M − H]^−^. ^1^H NMR (CD_3_OD) *δ*: 2.61 (2H, d, *J* = 7.5 Hz, H-2), 2.95 (2H, t, *J* = 7.5 Hz, H-3), 6.88 (1H, s, H-5), 7.28 (1H, s, H-8), 7.51 (1H, d, *J* = 2.1 Hz, H-9), 6.64 (1H, d, *J* = 2.2 Hz, H-10); ^13^C NMR (CD_3_OD) *δ*: 178.0 (C-1), 35.9 (C-2), 27.5 (C-3), 125.2 (C-3a), 154.6 (C-4), 98.3 (C-5), 156.2 (C-6), 121.0 (C-7), 122.2 (C-8), 144.6 (C-9), 107.2 (C-10). Compared with the data reported in the published paper [29], this compound was identified as 6,7-furano-hydrocoumaric acid.

**P-2**: C_12_H_12_O_4_, white powder. IR (KBr) *υ*_max_ 3416.77, 1734.91 cm^−1^. ESI MS *m*/*z* 219.15 [M − H]^−^. ^1^H-NMR (CD_3_OD) *δ*: 2.64 (2H, d, *J* = 7.8 Hz, H-2), 2.95 (2H, t, *J* = 7.8 Hz, H-3), 6.88 (1H, d, *J* = 0.9 Hz, H-5), 7.26 (1H, d, *J* = 0.9 Hz, H-8), 7.52 (1H, d, *J* = 2.2 Hz, H-9), 6.65 (1H, dd, *J* = 2.2 Hz, H-10), 3.64 (3H, s, OMe); ^13^C NMR (100 MHz, CD_3_OD) *δ*: 175.9 (C-1), 35.4 (C-2), 27.5 (C-3), 126.3 (C-3a), 154.6 (C-4), 98.2 (C-5), 156.2 (C-6), 121.2 (C-7), 122.2 (C-8), 144.6 (C-9), 107.1 (C-10), 52.0 (OMe). Compared with the data reported in the published paper [29], this compound was identified as 6,7-furano-hydrocoumaric acid methyl ester.

**IP-1**: C_11_H_10_O_4_, white powder. IR (KBr) 3423.79, 1053.94 cm^−1^. ESI MS *m*/*z* 205.10 [M − H]^−^. ^1^H NMR (CD_3_OD) *δ*: 2.60 (2H, d, *J* = 7.8 Hz, H-2), 2.96 (2H, t, *J* = 7.8 Hz, H-3), 6.92 (1H, d, *J* = 8.5 Hz, H-7), 7.03 (1H, d, *J* = 8.5 Hz, H-8), 7.56 (1H, d, *J* = 2.2 Hz, H-9), 6.91 (1H, d, *J* = 2.2 Hz, H-10); ^13^C NMR (CD_3_OD) *δ*: 177.8 (C-1), 35.9 (C-2), 26.6 (C-3), 121.1 (C-3a), 149.2 (C-4), 118.3 (C-5), 156.8 (C-6), 103.8 (C-7), 127.5 (C-8), 144.6 (C-9), 104.7 (C-10). Compared with the data reported in the published paper [30], this compound was identified as 5,6-furano-hydrocoumaric acid.

**IP-2:** Obtained as a white powder, the molecular formula was deduced to be C_11_H_12_O_4_, based on the quasi-molecular ion peak in the negative HR-ESI-MS at *m*/*z* 219.0656 ([M − H]^−^, calculated 219.0657). The IR spectrum displayed strong absorption bands at 1734.91 cm^−1^, indicating an ester group, and 3424.17 cm^−1^, suggestive of the hydroxyl function, and 1054.17 cm^−1^, suggestive of the propyl structure (C–C–C). The complete unambiguous assignments for the ^1^H and ^13^C NMR signals of IP-2 were made by a combination of the ^1^H−^1^H COSY, HSQC, HMBC, and nuclear overhauser effect spectroscopy (NOESY) spectra, suggesting the presence of a methoxy group at *δ* 3.64 (3H, s, OMe), one olefinic at *δ* 7.56 (1H, d, *J* = 2.2 Hz, H-9), 6.91 (1H, br d, *J* = 2.2 Hz, H-10). The other NMR spectral data of IP-2 are listed as below, namely: ^1^H NMR (CD_3_OD) *δ*: 2.63 (2H, d, *J* = 7.8 Hz, H-2), 2.97 (2H, t, *J* = 7.8 Hz, H-3), 6.91 (1H, br d, *J* = 8.4 Hz, H-7), 7.01 (1H, d, *J* = 8.4 Hz, H-8); ^13^C NMR (100 MHz, CD_3_OD) *δ*: 175.9 (C-1), 35.7 (C-2), 26.7 (C-3), 120.8 (C-3a), 149.2 (C-4), 118.2 (C-5), 156.9 (C-6), 103.7 (C-7), 127.5 (C-8), 144.7 (C-9), 104.7 (C-10), 52.2 (OMe). It is not difficult to find that the NMR spectral data of IP-2 were found be like IP-1, so we can reasonably conclude that IP-2 is the methyl-esterification product of IP-1. Thus, the structure of the new compound was unambiguously identified as 5,6-furano-hydrocoumaric acid methyl ester. The related spectrograms are listed in the supporting information (Figures S2–S8).

### 2.2. Method Validation of the Archetype and Metabolites

#### 2.2.1. UPLC-MS/MS Condition Optimization

The initial step was to optimize the precursor, product ions, and other parameters of six compounds and I.S. We found that P, IP, and I.S. presented a stronger ionization in the positive ion mode, while the other four metabolites presented a stronger ionization in the negative ion mode. The advantage of the instrument we have chosen is that it could analyze the samples in both the positive and negative mode simultaneously. Furthermore, the Q1 pre-bias, Q3 pre-bias, and collision energy (CE) were optimized to enhance the ion transmissions during the experiment. The parameters that were optimized are listed in Table 1. Multiple groups of the mobile phase, including MeOH–water and can–water, which contain different pH modifiers (ammonium acetate, formic acid, and ammonia), were tried to choose the optimal mobile phase system. Finally, a 1-mM ammonium acetate aqueous solution (A) and acetonitrile (ACN; B) were chosen as the mobile phase, which produced the best response and sensitivity. The gradient elution program is shown in Table S1, with the flow rate set as 0.35 mL/min, while the column temperature is 30 °C.

#### 2.2.2. The Assay Validation

**Figure 3:**
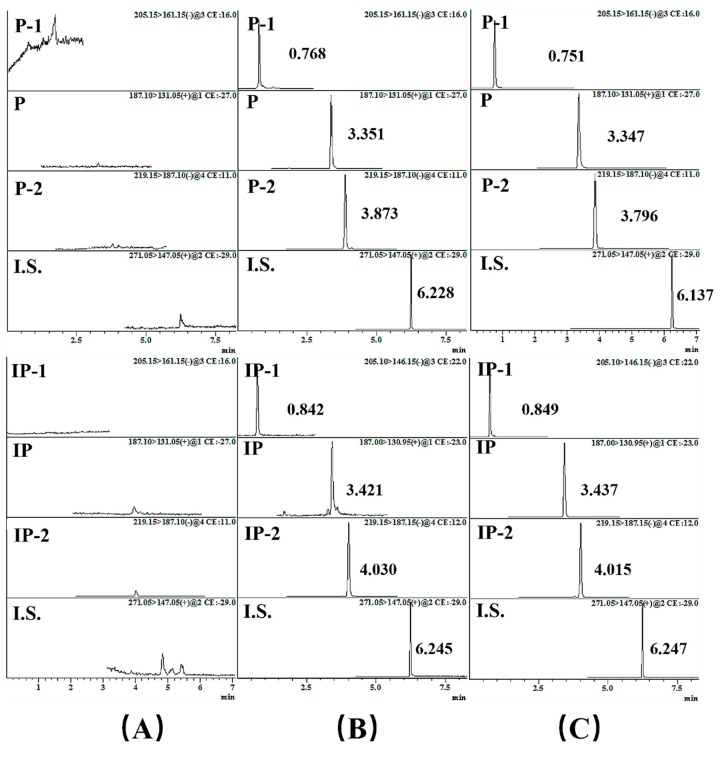
The typical MRM chromatograms with different disposal manners; (**A**) blank bacterial suspension; (**B**) blank bacterial suspension spiked with the six analytes and I.S. (LQC); (**C**) the chromatograms of the six components and I.S. in an active bacterial suspension, which were obtained at 12 h.

Two sets of chromatographic peaks of the three constituents and I.S. showed very good resolutions in Figure 3. The bacterial suspension samples show no interfering peaks from the endogenous substances. Figure 3 exhibits the representative multiple reaction monitoring (MRM) chromatograms of the blank heat-inactivated incubation solution (A); a spiked bacterial suspension sample with the components and the I.S. (LQC, B); and the sample obtained at 12 h after the biotransformation of P/IP (C). P, P-1, P-2, IP, IP-1, IP-2, and I.S. were eluted at 3.351, 0.768, 3.873, 3.421, 0.842, 4.030, and 6.245 min in an LQC sample, respectively.

As shown in Table 2, all the components presented a good linearity for all of the correlation coefficients (*r*^2^), which were ≥0.9906. In general, a detector of the signal to noise (S/N) ratio of 3:1 was denoted as the lower limit of detection (LLOD), and 10:1 as the lower limit of quantitation (LLOQ). The LLODs of P, P-1, P-2, IP, IP-1, and IP-2 were 2.24, 2.65, 1.01, 2.53, 2.23, and 1.07 ng/mL, respectively, and the LLOQs of P, P-1, P-2, IP, IP-1, and IP-2 were 4.88, 5.30, 2.02, 5.06, 4.46, 2.14 ng/mL, respectively, which were suitable as the thresholds for the quantitation of the components.

As shown in Table 3, the results of the precision and accuracy indicated that the established method is satisfactory to analyze the six components in the human intestinal bacteria incubation system. The intraday and interday precision (Relative Standard Deviation; RSD%) of all of the QC samples were less than 13.69%, and the accuracies (RE%) ranged from −10.26% to 12.43%. 

The extraction recovery and matrix effects experiments also have been established. The extraction recoveries were within the range of 82.04% ± 8.58%–98.24% ± 12.54%. The matrix effects of all of the compounds were acceptable. with the values of 83.88% ± 14.10%–110.48% ± 10.38% in the heat-inactivated incubation solution (Table 4). Liquid–liquid extraction with EtOAc was a workable method for the analysis of the components and the I.S. The extraction recoveries and the matrix effects of I.S. were 87.75% ± 12.45% and 87.89% ± 5.89%, respectively.

The stability of the six analytes was investigated under the given conditions, illustrated in Table S3 in the supporting information. The concentrations obtained ranged from −14.41% to 14.61% of their nominal concentrations, which suggests that the analytes were stable in the heat-inactivated incubation solution after storage for 4 h at an ambient temperature and being kept at 4 °C for 12 h.

### 2.3. Time Course of the Biotransformation of P and IP by Human Intestinal Flora

**Figure 4:**
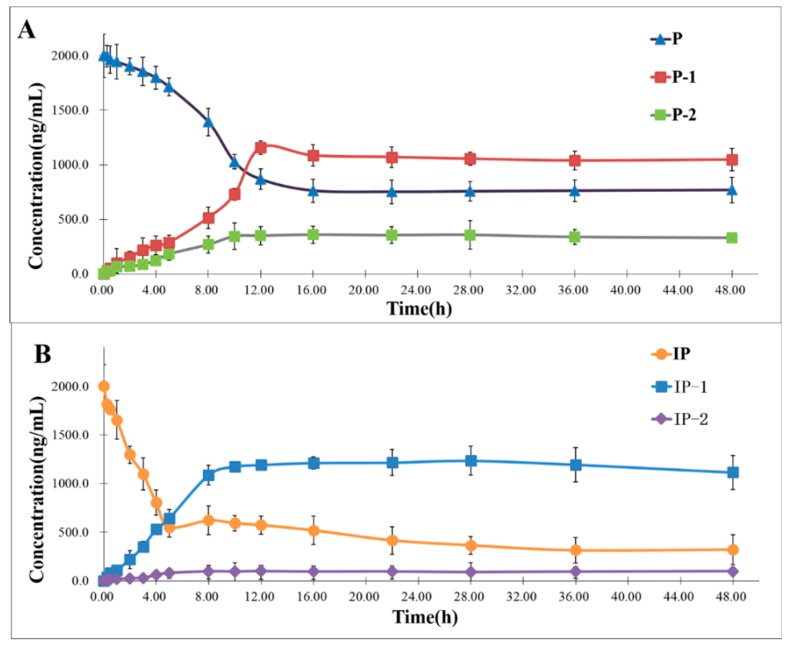
The time course of biotransformation of P (**A**) and IP (**B**) by active human intestinal flora (*n* = 3, mean ± standard deviation (SD).

Figure 4A,B shows the time course of the biotransformation of P and IP at a dose of 0.15 mg/mL, respectively. Because of the similar metabolic pathway of P and IP, we used P as an example to discuss the metabolic process of P and IP incubated with human fecal microflora. The results indicated that P was almost completely consumed 12 h after the start of incubation, and the other biotransformation products were dynamic generation. The ring structure of P depends on the ester bond, which could easily break in a strong environment, such as the harsh alkali conditions and the metabolism, by digestive enzymes from the intestine. As a result, P could be further metabolized by ring splitting to form a further product, P-1. The hydroxy phenylacetic acid derivative of P increased in the first 8 h, and was then maintained smoothly. The product P-2 was produced gradually with a much lower conversion than P-1, which suggests that P-1 could be a biotransformation intermediate. Moreover, P-2 is the methylate of P-1, which could also have been available to support this viewpoint. After about 12 h of incubation, P changed more and more slowly, and finally it tended to steady. Then, after 16 h, the conversion degree of all of the analytes started to level off. Furthermore, the transformation efficiency of P was a little more than IP, which might be related to the compounds′ steric configuration.

### 2.4. MTT Assay

**Figure 5:**
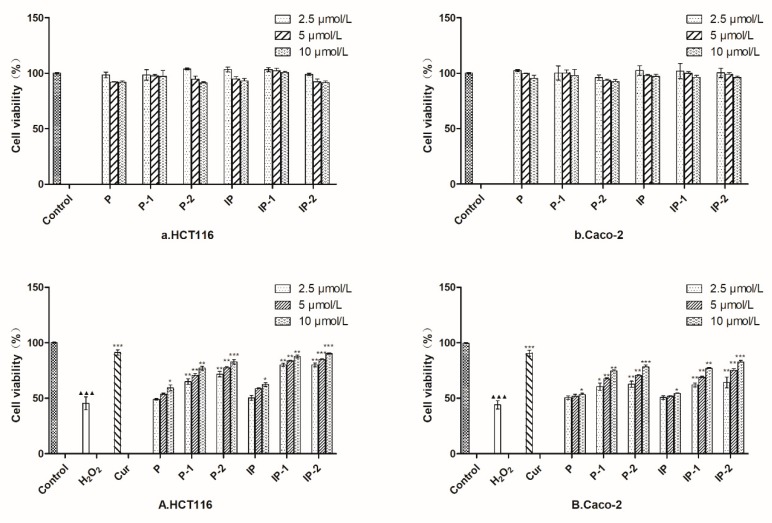
Cytotoxicity ((**a**) for HCT116 cell line; (**b**) for Caco-2 cell line) and the protect effects against H_2_O_2_-induced oxidative damage ((**A**) for HCT116 cell line; (**B**) for Caco-2 cell line) of P, IP, and their transformation products. The Cur group was the positive control group. Data are shown as the mean ± SD (*n* = 3). ^▲▲▲^ *p* < 0.001 vs control group; * *p* < 0.05, ** *p* < 0.01, *** *p* < 0.001 vs the H_2_O_2_ group.

As shown in Figure 5A,B, the pretreatment of heterogeneous human epithelial colorectal adenocarcinoma cells (Caco-2) and human colorectal cells (HCT116) cells for 24 h with the co-culture of six analytes (2.5, 5, and 10 μM) did not exhibit a cytotoxicity contrasted with the control cells, showing that the concentrations we chose did not injure the cell vitality during the process of the co-culture.

### 2.5. Compounds Alleviate the Decreased Viability of HCT116 and Caco-2 Cells Induced by H_2_O_2_

**Figure 6:**
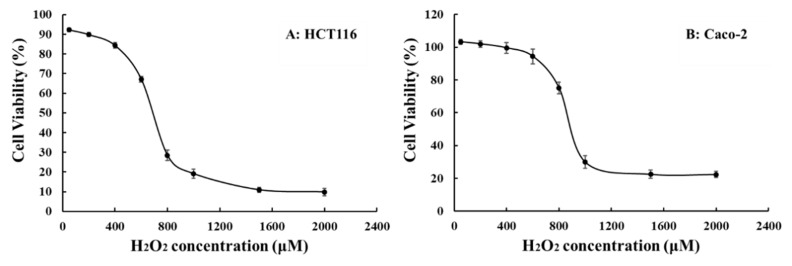
The cell viability of different cell lines against the H_2_O_2_ concentration ((**A**): human colorectal cells (HCT116); (**B**): heterogeneous human epithelial colorectal adenocarcinoma cells (Caco-2)).

The damage of H_2_O_2_ on the HCT116 and Caco-2 cells was analyzed, and the concentration vs cell viability (CV) relation graphs are shown in Figure 6A,B, respectively. However, after H_2_O_2_ exposure, the CV was significantly decreased (*p* < 0.001) vs the control, at a concentration of 700 μM (43%) for HCT116 and 900 μM (47%) for Caco-2, which were the concentrations chosen as the model concentration. As shown in Figure 5A,B, P and IP have no obvious activities, while the biotransformation products of P and IP could be remarkable for improving the cell injury induced by H_2_O_2_. Take IP-2 for example, in the HCT116 cell line, it could increase the CV to 79%, 84%, and 89% at 2.5, 5, 10 μM, respectively. In Caco-2 cell line, it could increase the CV to 62%, 74%, 82% at 2.5, 5, and 10 μM, respectively. All the four biotransformation products (P-1, P-2, IP-1, and IP-2) could obviously resist the cell damage in a dose-dependent way. The Cur group (10 μM) was the positive control group, which could increase the CV to around 90% for the HCT116 and Caco-2 cell lines. The median effective dose (ED50) values were also calculated using SPSS statistics package v.20.0 (SPSS Inc., Chicago, USA). The ED50 values of P, P-1, P-2; IP, IP-1, and IP-2 were 18.08 ± 4.32, 8.54 ± 3.14, 7.78 ± 0.11; 17.09 ± 1.55, 5.63 ± 0.55, and 3.49 ± 0.11 μM, respectively. The ED50 value of Cur is 3.801 ± 0.66 μM.

### 2.6. Discussion

According to the chemical structures of the archetypes and their metabolites, tied together with the time–course curve, the main pathways of P and IP metabolism by human intestinal flora could be speculated in detail. As illustrated in Figure 2, the metabolism of the linear-type furanocoumarin P by human intestinal flora suggested that the main pathway involved a reduction at the α,β-unsaturated lactone ring of the coumarin skeleton and methylation [29]. In the first step, the human intestinal flora was known to metabolize P by a reductive mechanism, leading to the occurrence of P-1. As for P-2, it might be formed from the step of the esterification of P-1, by related enzymes from the intestinal bacteria. As the isomer of P (linear-type furanocoumarin), the conversion process of IP (angle-type furanocoumarin) was basically the same in the human intestinal flora, which is described in Figure 2. Compared with the time course of IP, we could find that the holistic reaction rate of P could to be relatively higher with the larger amount of transformation products and the longer reaction time. As shown in the time courses, the reduction and methylation reactor all tend to be stable after 8 h for IP, and 12 h for P. The concentration of IP-2 was just under the concentration of P-2, and while admitting more research is needed, we still think the structural differences between the isomers might account for these differences.

The pure products we obtained from the microbial transformed processes could be used for further pharmacological evaluation. As for the pharmacological activities′ studies, PC has been discovered to have significant antioxidant, anticancer, and estrogenic activities. According to TCM, for hundreds of years, the seeds of PC have often been chosen as a main force in several formulas for treating IBD. It is well known that the overproduction of reactive oxygen species could incur oxidative stress, which plays a key role in the formation of IBDs. Treatment with H_2_O_2_ could induce oxidative stress and a degree of oxidative damage in Caco-2 cells [31] and HCT116 cells [32]. According to the relationship between the H_2_O_2_ levels and the survival rates of HCT116 and Caco-2 cell lines, the injured concentrations were chosen at 700 μM and 900 μM in this research, respectively. Guo et al. [33] evaluated the antioxidant activities of six compounds, including psoralidin, from PC extract, which exhibited strong antioxidant activities; however, P and IP do not possess any antioxidant activities either, which is consistent with the results of our experiments. Also, our research revealled that P-1, P-2, and IP-1, IP-2 had stronger antioxidant stress effects than coumarins (P and IP) [34]. To our best knowledge, there are very few studies about the metabolites of P and IP; through the research about the microbial transformation of P, which was performed with the fungus *Glomerella cingulate*, Shinsuke et al. [29] obtained P-1 for the first time, and the β-secretase inhibitory activity of P-1 and P-2 in vitro were proceeded. P-2 was shown to have a β-secretase inhibitory activity, with an IC_50_ value of 0.84 ± 0.06 mM. The bioactivities of IP-1 and IP-2 were first researched in our issue. Multiple literatures have reported that the metabolites of TCM performed stronger pharmacological activities than the original components, which are the final active compounds with the greatest importance [35,36]. Furthermore, the bioactivities of P and IP, especially their metabolites, exhibited great valuable to research among the topic of IBD.

## 3. Experimental Section

### 3.1. Material and Conditions

#### 3.1.1. Material and Reagents

P and IP with 98% purity (Figure 1) were isolated from the fruits of PC by our research group; some of the chemical information are listed in the supporting information (Figures S9–S14), which were identified by the techniques of IR, MS, 1H-NMR, 13C-NMR, and HPLC analysis, as well as in comparison with the spectral data reported in the literature [37]. Isoimperatorin with 98% purity was used as an internal standard (I.S.; the structure illustrated in Figure 1) was isolated from the roots of *Angelica dahurica* cv. *Yubaizhi* by our research group [38], which was identified by the techniques of IR, MS, ^1^H-NMR, ^13^C-NMR, and HPLC analysis. GAM (general anaerobic medium) was obtained from Beijing Land Bridge Technology Co., Ltd. (Beijing, China). LC-MS grade acetonitrile (ACN), methanol (MeOH), and formic acid were purchased from Fisher Chemical (Fair lawn, NJ, USA). HPLC-grade ethyl acetate (EtOAc) and n-butanol (n-BuOH) were obtained from Beijing Chemical Works (Beijing, China). Deionized water was purified on a Milli-Q water system (Bedford, MA, USA). All the other chemicals and reagents we used were analytical grade.

Dimethyl sulfoxide (DMSO), curcumin (Cur), hydrogen peroxide solution (H_2_O_2_), (3 wt % in H_2_O), and 3-(4,5-dimethyl-2-thiazolyl)-2,5-diphenyl-2H-tetrazolium bromide (MTT), were purchased from Sigma-Aldrich (St. Louis, MO, USA). The 96-well plates and cell culture dishes were purchased from Corning Inc. (Lowell, MA, USA). Dulbecco′s modified Eagle′s medium (DMEM), fetal bovine serum (FBS), trypsin, and penicillin-streptomycin solution were purchased from the Gibco^®^ Life Technologies Incorporated Company, (Grand Island, NY, USA).)

#### 3.1.2. Instrumental and UPLC-MS/MS Conditions

The one- and two-dimensional (1D and 2D) NMR spectra was analyzed on a Bruker AVANCE III 400 spectrometer (Fällanden, Switzerland), where the tetramethylsilane (TMS) was used as an internal standard with CD_3_OD as the solvent. All the cultures in this study were carried out under anaerobic conditions, using the Thermo Scientific 1029 Forma Anaerobic System using Oxoid BR0055 as the anaerobic indicator.

The UPLC experiment was carried out on a Shimadzu UPLC instrument equipped with an 8050 series mass spectrometer with the electrospray ionization interface (Shimadzu, Kyoto, Japan). The separation was achieved on a Kinetex^®^ C_18_ column (100 mm × 2.1 mm, 2.6 μm) equipped with a Kinetex^®^ RP_18_ guard column (30 mm × 2.1 mm, 2.6 μm) at a flow rate of 0.35 mL/min, analyzed with a gradient elution for 7 min. The UPLC mobile phases consisted of (A) a 1-mM ammonium acetate solution and (B) ACN. The binary gradient program is presented in the Table S1. The column temperature was set at 30 °C and the injection volume was 2 μL. Six components and the I.S. were analyzed using multiple reaction monitoring (MRM) modes in the positive (for P and IP) and negative (for four transformation products) ionization mode, simultaneously. The other parameter settings after optimization were as follows: DL temperature of 250 °C, drying gas flow rate of 10 L/min, heat block temperature of 400 °C, interface temperature of 300 °C, and nebulizing gas flow of 3 L/min. The gases used were high purity nitrogen (99.99%). The monitoring transitions and relative MS parameters are presented in Table 1.

### 3.2. Preparation of Representative Human Gut Bacteria

This study was authorized and supervised by the Ethics Committee of Peking University Health Science Center, Peking University. All the procedures involving human participants conformed to the ethical standards of the committee. The fresh human feces sample was offered by four healthy Chinese volunteers (two females and two males, 22–30 years old), with at least three months without antibiotics use or gastrointestinal diseases. The feces we collected were immediately premixed and homogenized anaerobically; four grams of fresh feces were suspended with 100 mL of the GAM broth. We strained the fecal suspensions through nine pieces of gauze, while the subsequent filtrates were incubated in an anaerobic incubator under an anaerobic environment (H_2_ 5%, CO_2_ 10%, and N_2_ 85%) in vitro at 37 °C.

### 3.3. Elucidation of the Biotransformation Products from P and IP

Then, 46 g GAM broth was dissolved in 1000 mL of water; the ultrasonic dissolving method was used to make the solution dissolves adequately. The pH was adjusted to 7.3 before autoclaving at 121 °C for 20 min. After being cooled to 45 °C, 6 mg hematin chloride and 1 mg vitamin K were added in the broth. Then, 10 mL of activated bacterial flora were added to GAM solution (1000 mL) and cultivated for 12 h, and 500 mg of P or IP were added, respectively. The floras were cultivated at 37 °C for 48 h in an anaerobic environment, and the cultured suspension was inactivated by being extracted with 2000 mL EtOAc three times. The combined EtOAc layer was evaporated, and the residue was dissolved by MeOH. The extract was simply separated and purified by Sephadex LH–20, by eluting with MeOH to obtain several fractions. The fractions were determined by HPLC, and some fractions containing the target compounds were injected into the semi-preparative reversed phase HPLC m while the mobile phase we used was a 30% ACN aqueous solution containing 0.01% formic acid.

### 3.4. Time Courses of the Biotransformation of P and IP by Human Intestinal Flora

The biotransformation of P and IP by human intestinal bacteria was performed in 10-mL incubation glass tubes containing 3 mL GAM and 200 µL of the intestinal microflora solution, and cultivated for 12 h. The standard solutions of P and IP were prepared by dissolving accurately weighted P and IP in DMSO, for which the final concentrations were 0.15 mg/mL for both. Then, 15 µL of P and IP DMSO solution were added, for which the added volumes of DMSO were no more than 0.5% of the total incubation system [39]. The biotransformation was stopped at 0.25, 0.5, 1, 2, 3, 4, 6, 8, 10, 12, 16, 24, 30, 36, and 48 h. A GAM sample without a microflora solution injecting was regarded as the control, and a microflora solution without the compounds was regarded as the blank. Also, the sample was added in the inactivated bacteria liquid to rule out the effect of the culture medium. All of the experiments were performed in triplicate for each time point, which were processed according to the method described mentioned above. Briefly, 50 µL I.S. (2000 ng/mL) was pipetted into the bacteria solution, then extracted with 6 mL EtOAc three times. Each organic layer was carefully transferred to a clean 10 mL EP tube and dried at 40 °C under a gentle flow of N_2_ gas, and the residue was re-dissolved with 100 µL mobile phase by a vortex for 1 min, then centrifuged at 15,000 rpm for 10 min. The supernatant was filtered with a 0.22 µm filter, and two microliters of the subsequent filtrate were injected into the detective system.

### 3.5. Preparation of Standards and Quality Control Samples

All the prepared solutions were prepared in MeOH, and two sets of mixed stock solution were obtained (2440, 2650, and 1010 ng/mL for P, P-1, and P-2, and 2530, 2230, and 1070 for IP, IP-1, and IP-2). Then, the solutions further diluted in a pattern of 1:2:2:2.5:4:5:2.5 were applied to generate a series of concentrations of working solutions, which were kept at −4 °C. The I.S. was prepared with MeOH at a concentration of 2000 ng/mL. All of the calibration standards and three levels of the quality control (QC) samples (12.20, 610.0, and 1952 ng/mL for P; 13.25, 662.5, and 2120 ng/mL for P-1; 5.05, 252.5, and 808 ng/mL for P-2; 12.65, 632.5, and 2024 ng/mL for IP; 11.15, 557.5, and 1784 ng/mL for IP-1; and 5.35, 267.5, and 856 ng/mL for IP-2 in a drug-free culture medium were prepared by spiking the inactivated bacterial suspensions and relative mixed standard working solutions into the blank culture medium. The linearity ranges were 4.88–2440 ng/mL for P, 5.30–2650 ng/mL for P-1, 2.02–1010 ng/mL for P-2, 5.06–2530 ng/mL for IP, 4.46–2230 ng/mL for IP-1, and 2.14–1070 ng/mL for IP-2, respectively.

### 3.6. Evaluation of Cytotoxicity

The CV was estimated by the MTT assay [40] against HCT116 (human colorectal cells) and Caco-2 (heterogeneous human epithelial colorectal adenocarcinoma cells) cell lines. After the incubation of HCT116 and Caco-2 cell lines at a density of 2 × 10^5^ cells/mL on 96-well plates for 24 h (Caco-2 was incubated for 36 h), the cells were cultured under several test concentrations of compounds for 24 h. Then, 20 μL of a MTT test solution (5 mg/mL) was added to each well and incubated for 4 h. The formazan was fully dissolved by 100 μL DMSO and the OD (average optical density) values were detected by a microplate reader (Multiskan Mk3, Thermo) at 490 nm. with a 650 nm reference wavelength. The tests were performed separately three times. The cell viabilities were calculated as the percent account for the control group as the following equation:

Cell viability (%) = [(*A*_H2O2_ − *A*_blank_)/(*A*_contral_ − *A*_blank_)] × 100% (1)

### 3.7. Alleviating Oxidative Stress Caused by H_2_O_2_ in HCT116 and Caco-2 cells

The alleviating oxidative stress effects of six compounds against two cell lines were conducted, and the oxidative stress model was established by the H_2_O_2_. The cells were cultured in 96-well plate as described in the above-mentioned, which were the deal with several test concentrations of H_2_O_2_ for 6 h to explored optimal concentration. The CV was detected by the MTT test. After incubating the cells according to the method mentioned above, the DMEM medium was discarded and treated with 100 μL of FBS-free DMEM medium containing different concentrations (2.5, 5, and 10 μM) of six compounds for 2 h. Then, the H_2_O_2_ was added into each well (700 μM for HCT116, and 900μM for Caco-2) and incubated for 6 h. The control group contained FBS-free DMEM, and no H_2_O_2_ was added to the cells, whereas the FBS-free DMEM without cells or H_2_O_2_ in blank wells. The cells were treated with H_2_O_2_ and Cur (10 μM) as a positive control group. All of the CVs were detected by the MTT assay mentioned above. All of the experiments were conducted for three replicate wells in three parallel experiments.

### 3.8. Statistical Analysis

The data were calculated using SPSS statistics package v.20.0 (SPSS Inc., Chicago, IL, USA) and GraphPad Prism (GraphPad software Inc., San Diego, CA, USA). The results are showed as the mean ± standard deviation (SD), and the differences between the test group and the control group were analyzed by Student′s *t*-test, for which the results were recognized significant at * *p* < 0.05, ** *p* < 0.01, and *** *p* < 0.001.

## 4. Conclusions

The research demonstrated that human intestinal bacteria transformation is an essential part of the metabolism of P and IP in vivo, for which the reduction reaction at the α, β-unsaturated lactone ring of the coumarin skeleton and methylation are the main pathways. The results demonstrated that the antioxidant stress activity of PC may rely on the metabolism of P and IP by intestinal bacteria for the greater activity of the metabolic products. However, further studies should be done in order to illustrate the mechanism of action of the pharmacological activity of IBD.

## Figures and Tables

**Table 1:** Optimized multiple reaction monitoring (MRM) parameters of five compounds and internal standard (I.S.) in mass detection.

Analyte	MRM	Precursor Ion–Product Ion (*m*/*z*)	Q1 Pre-Bias ^*^ (V)	Collision Energy (V)	Q3 Pre-Bias ^*^ (V)	Dwell Time (msec)	Retention Time (min)
P	MRM (+)	187.10–131.05	−14.0	−27.0	−23.0	100.0	3.351
P-1	MRM (−)	205.15–161.15	23.0	16.0	16.0	100.0	0.768
P-2	MRM (−)	219.15–187.10	16.0	11.0	18.0	100.0	3.873
IP	MRM (+)	187.00–131.95	−14.0	−23.0	−22.0	100.0	3.421
IP-1	MRM (−)	205.10–146.15	15.0	22.0	28.0	100.0	0.842
IP-2	MRM (−)	219.15–187.15	16.0	12.0	19.0	100.0	4.030
I.S.	MRM (+)	271.05–147.05	−30.0	−29.0	−27.0	44.0	6.245

*: Q1 pre-bias and Q3 pre-bias: voltage enhance the ion transmission of the precursor ion and the product ion; dwell time: residence time during an acquisition point.

**Table 2:** The regression data, lower limit of quantitations (LLOQs), and lower limits of detections (LLODs) of six compounds in human intestinal flora.

Analytes	Regression Equation	*r* ^2^	Linear Range *	LLOQ *	LLOD *
P	y = 46.4226x + 198.909	0.9977	4.88–2440	4.88	2.44
P-1	y = 318.924x + 316.47	0.9921	5.30–2650	5.30	2.65
P-2	y = 48.9102x + 1577.72	0.9931	2.02–1010	2.02	1.01
IP	y = 227.105x − 2813.5	0.9939	5.06–2530	5.06	2.53
IP-1	y = 226.079x − 1952.0	0.9906	4.46–2230	4.46	2.23
IP-2	y = 854.015x − 2543.2	0.9957	2.14–1070	2.14	1.07

*: The linear range, LLOQ and LLOD units of compounds in human intestinal flora are ng/mL.

**Table 3:** Precision and accuracy of the determination of six compounds in the human intestinal flora (*n* = 18, six replicates per day for three days).

Compounds	Spiked Concentration (ng/mL)	Measured Concentration (ng/mL)	Accuracy (%)	Precision (%)	
				Intra-Day	Inter-Day
P	4.880	4.80 ± 0.50	−1.40	4.80	9.50
	12.20	13.22 ± 1.70	8.39	12.72	13.69
	610.0	611.0 ± 75.17	0.16	13.05	3.04
	1952	2067 ± 144.0	5.89	7.14	5.47
P-1	5.300	5.140 ± 0.330	−3.00	7.80	5.20
	13.25	13.07 ± 1.530	−1.36	12.35	3.63
	662.5	594.5 ± 60.34	−10.26	10.11	10.45
	2120	2204 ± 203.0	3.96	9.19	9.38
P-2	2.020	2.240 ± 0.156	10.80	3.50	6.30
	5.050	5.680 ± 0.590	12.43	10.56	9.82
	252.5	250.8 ± 29.33	−0.69	12.00	9.08
	808.0	847.3 ± 34.10	4.86	4.25	1.54
IP	5.060	5.140 ± 0.321	1.60	4.40	5.60
	12.65	12.65 ± 0.460	0.12	3.23	5.99
	632.5	556.7 ± 63.88	−0.14	11.54	10.96
	2024	1794 ± 110.1	0.57	8.45	2.78
IP-1	4.460	4.471 ± 0.407	8.20	9.60	7.00
	11.15	12.36 ± 1.041	−2.12	8.00	11.10
	557.5	528.0 ± 45.88	−5.30	8.65	9.01
	1784	1820 ± 86.62.3	2.02	6.49	7.85
IP-2	2.140	2.401 ± 0.321	12.20	9.20	11.80
	5.350	5.030 ± 0.430	−5.90	8.81	4.86
	267.5	242.2 ± 25.68	−7.75	10.93	7.79
	856.0	839.2 ± 65.35	−2.02	9.29	13.04

**Table 4:** Matrix effects and extraction recovery for the analytes in human intestinal flora (*n* = 6).

Compounds	Spiked Concentration (ng/mL)	Matrix Effect		Extraction Recovery	
		Mean (%)	RSD (%)	Mean (%)	RSD (%)
**P**	12.20	86.87	5.68	98.24	12.54
	610.0	88.37	7.94	95.99	5.57
	1952	98.49	11.52	91.86	5.12
P-1	13.25	89.77	3.54	85.82	8.49
	662.5	101.14	9.56	94.81	4.10
	2120	99.26	10.55	89.49	13.53
P-2	5.05	110.48	10.38	97.17	6.51
	252.5	96.99	4.52	96.02	4.84
	808	86.87	4.77	90.48	6.39
IP	12.63	91.15	10.80	95.39	4.67
	632.5	95.51	3.37	91.31	4.72
	2024	92.37	4.18	89.94	2.75
IP-1	11.15	94.00	7.38	89.65	3.87
	557.5	93.53	7.86	87.95	9.78
	1784	98.19	8.75	82.04	8.58
IP-2	5.35	98.55	11.08	97.28	13.34
	267.5	95.74	14.34	85.71	12.70
	856.0	83.88	14.10	94.07	14.67

## Supplementary Materials

The following are available online. Full IR, MS, and NMR spectra for compound IP-2, and other supplementary information of this research.
