# Supplementary material for: Transformation of Psoralen and Isopsoralen by Human Intestinal Microbial In Vitro, and the Biological Activities of Its Metabolites
Source: Molecules. 2019 Nov 12;24(22):4080. doi: 10.3390/molecules24224080 (PMC6891621; doi:10.3390/molecules24224080)
Supplement: Supplementary file 1 [file molecules-24-04080-s001.pdf]

Molecules **2017**, 22, x; doi: FOR PEER REVIEW

Supplementary Material:

Transformation of Soralen and Isopsoralen by Human Fecal Microbial In Vitro and Biological Activities of its Metabolites

Lu Liu [1], Lei Zhang [1], Ze-xu Cui [1], Xiao-yan Liu [1], Wei Xu [1], and Xiu–Wei Yang [1,*]

Procedures and Conditions of Chemical Separation

To simply purify the total transformation products, we used Sephadex LH–20 as the stationary phase, while MeOH was used as the moving phase. For the primary qualitative research, several fractions were injected into an Agilent 1290 UPLC system (Agilent Technologies, Waldbronn, Germany), equipped with a binary pump, an online vacuum degasser, an autosampler, and a thermostatically controlled column compartment. Then, the further isolation of the transformation products was achieved on a Beijing CXTH 3000 system (Beijing, China) equipped with an A1359 manual injector with a loop of 5 mL and a UV3000 UV detector. The LC Workstation was CXTH 3000 Chrome software, and a preparative Phenomenex Prodigy C_{18} column (250 × 21.2 mm, 5 μm; Phenomenex, Torrance, CA, USA) equipped with a C_{18} guard column (8 × 4 mm i.d., 5 μm; Dikma, China) was used for the isolation and purification of the compounds.

Method Validation

The linearity was calculated by plotting the peak area ratio (y) of the analytes to the I.S. vs the concentrations of seven standard samples (x), with a weighted factor (1/x2) to correct the bias. The minimum concentration point of the calibration

curve was defined as the lower limit of quantification (LLOQ) with acceptable accuracy (20 %, RSD) and precision (±20%, RE), with an S/N more than 10:1.

Three concentrations of QC samples (low, medium, and high), which were performed in an inactivated intestinal flora incubation solution, were used to assess the precision and accuracy of this method three different days ($n = 6$). The precision was defined as the RSD% of the measured concentration compared with the QC samples should be within 15% and 20% deviation for LLOQ. The accuracy was defined as RE% ±15% of the QC samples, except at the LLOQ where 20% was acceptable.

Three concentrations of QC samples were determined as well as the extraction recoveries, by comparing the peak areas of the blank substrates spiked before and after extraction ($n = 6$). Three concentrations of QC samples determined the matrix effects by comparing the peak areas of the samples spiked after being extracted with the same amount of standard solution in MeOH. The same method was also applied to the I.S. (2000 ng/mL), and all of experiments were carried out in six replicates.

The stability of six components, including room temperature stability (storage at the normal temperature for 4 h) and post-preparation stability (storage after sample preparation at 4 °C for 12 h) were evaluated in the intestinal flora incubation solutions at three QC levels with six replicates.

Table S1. Gradient elution program of the UPLC-MS/MS system.

Time(min)	A%	B%
0–1	70	30
1–2	60	40
2–4	60	40
4–5.5	10	90
5.5–7	10	90
7–7.1	70	30

(A) 1-mM ammonium acetate aqueous solution; (B) acetonitrile.

Table S2. Gradient elution program of HPLC-DAD system.

Time(min)	A%	B%
0	95	5
0–10	70	30
10–15	55	54
15–40	40	60
40–50	5	95
50–55	95	5

(A) 0.1% formic acid aqueous solution; (B) acetonitrile.

Table S3. Stabilities of the analytes in the blank heat-inactivated incubation solution (*n* = 6).

Compounds	Spiked CONC (ng/mL)	Stability (%RE)	
		Short-term	Post-term
	12.20	7.96	14.61
	610.0	4.99	−1.94
P	1952	4.61	12.60
	13.25	3.03	−5.51
	662.5	−14.41	−4.52
P-1	2120	7.10	7.47
	5.05	11.23	5.95
	252.5	−4.28	5.83
P-2	808.0	7.77	2.27
	12.63	5.66	0.24
	632.5	−14.01	−12.82
IP	2024	−10.99	−10.82
	11.15	11.60	−0.63
	557.5	−8.47	−1.82
IP-1	1784	5.14	3.28
	5.35	−0.09	−12.90
	267.5	−12.27	−9.85
IP-2	856.0	10.73	14.17

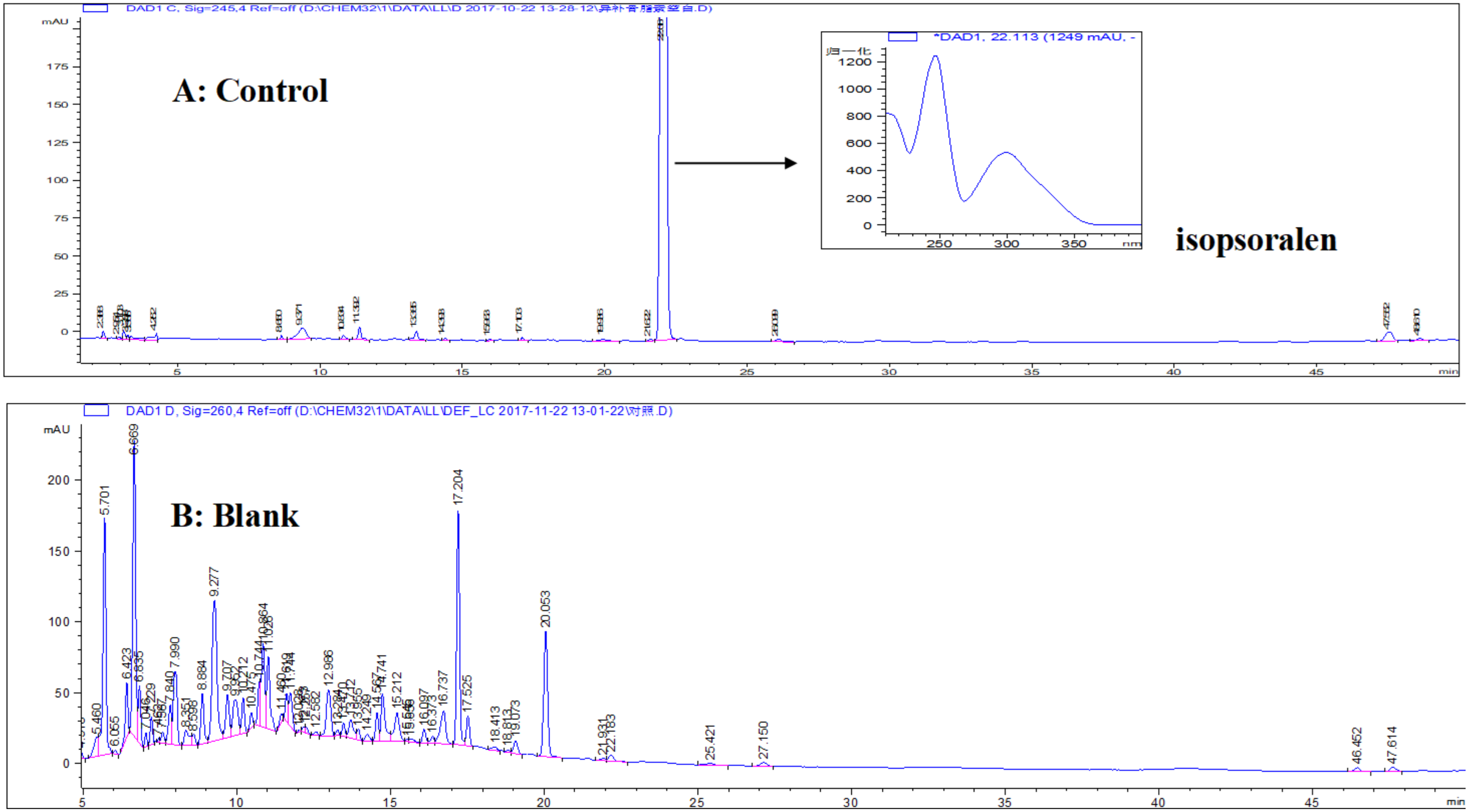
DAD1 C, Sig=245,4 Ref=off (D:\CHEM32\1\DATA\LL\D 2017-10-22 13-28-12\异补骨脂素空白.D)
A: Control
*DAD1, 22.113 (1249 mAU, -
isopsoralen
DAD1 D, Sig=260,4 Ref=off (D:\CHEM32\1\DATA\LL\DEF_LC 2017-11-22 13-01-22\对照.D)
B: Blank
mAU
min
nm

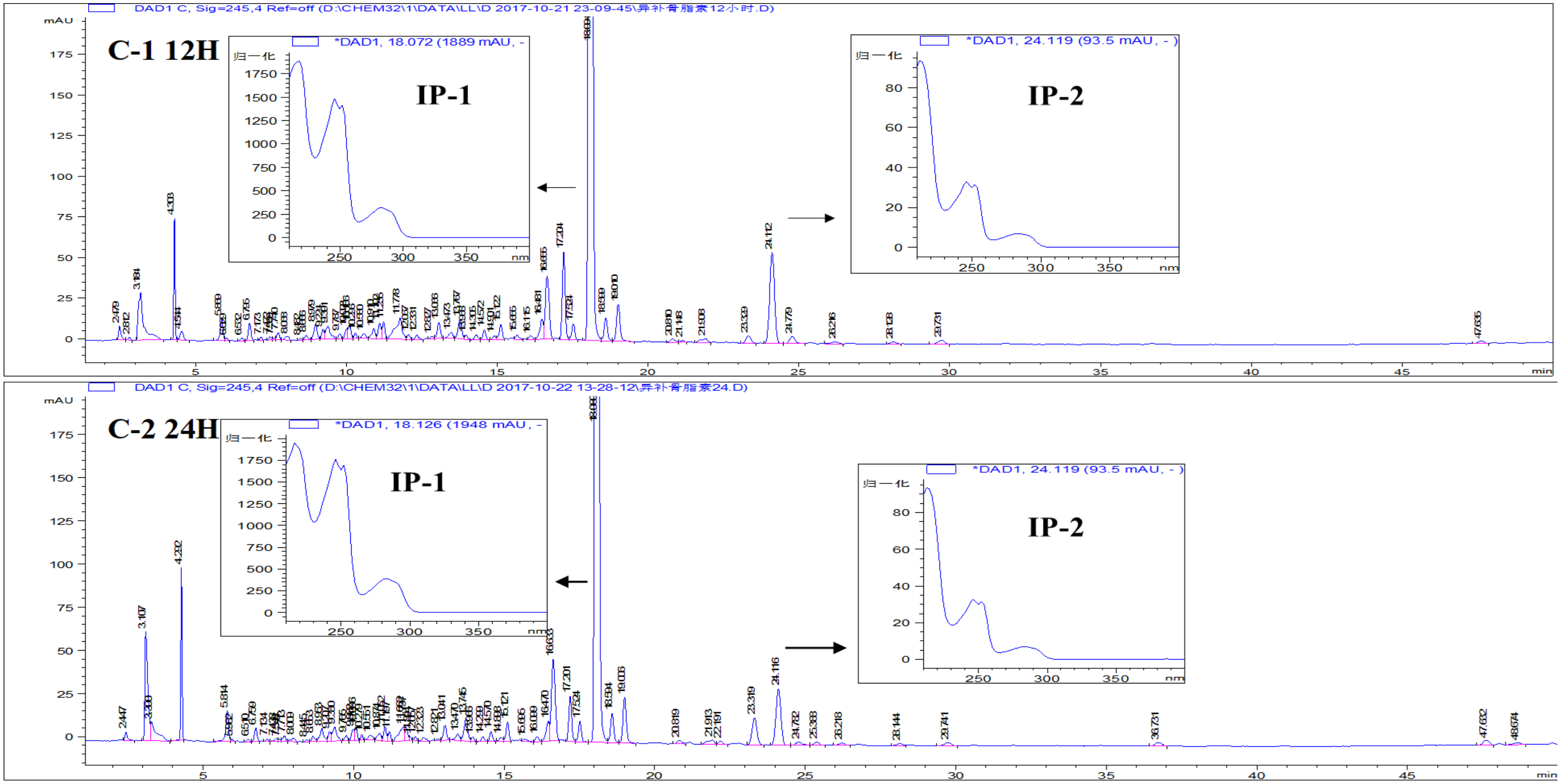

Figure S1. Typical HPLC chromatograms of the biotransformation of IP by human intestinal flora; ((A) control; (B) blank; (C-1) cocultivation for 12 h; (C-2) cocultivation for 24 h; where (A) and (C) were detected at 245.4 nm, and (B) was detected at 260.4 nm;). As shown in Figure S1, compared with the sample that only contained GAM and IP ((A) control sample) and incubation solution without analyses ((B) blank sample), tow metabolites from IP were obtained (C-1 for 12 h, and C-2 for 24 h). The metabolic pathway of P resembled that of IP.

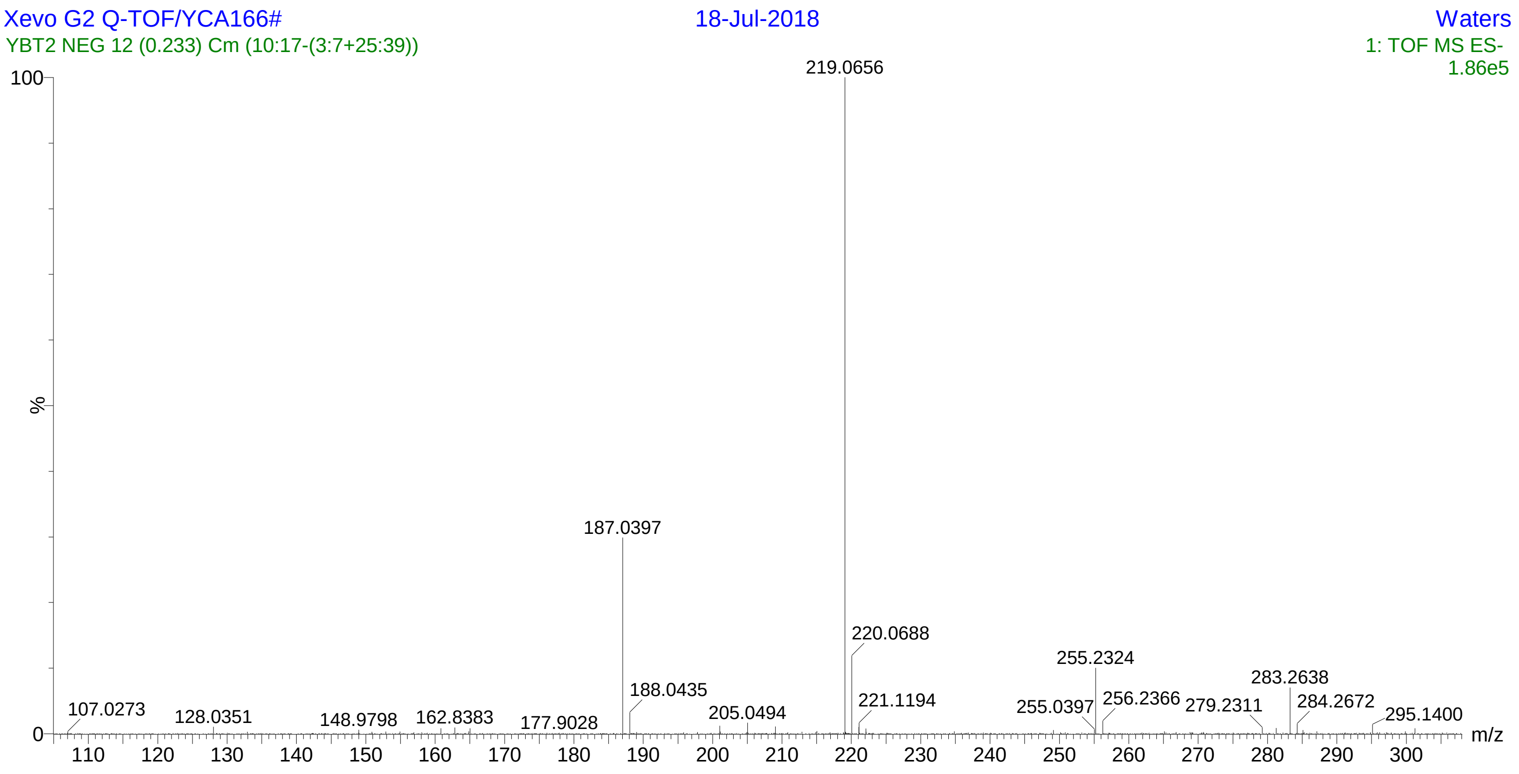

Figure S2. the HR-ESI-MS spectrum of IP-2.

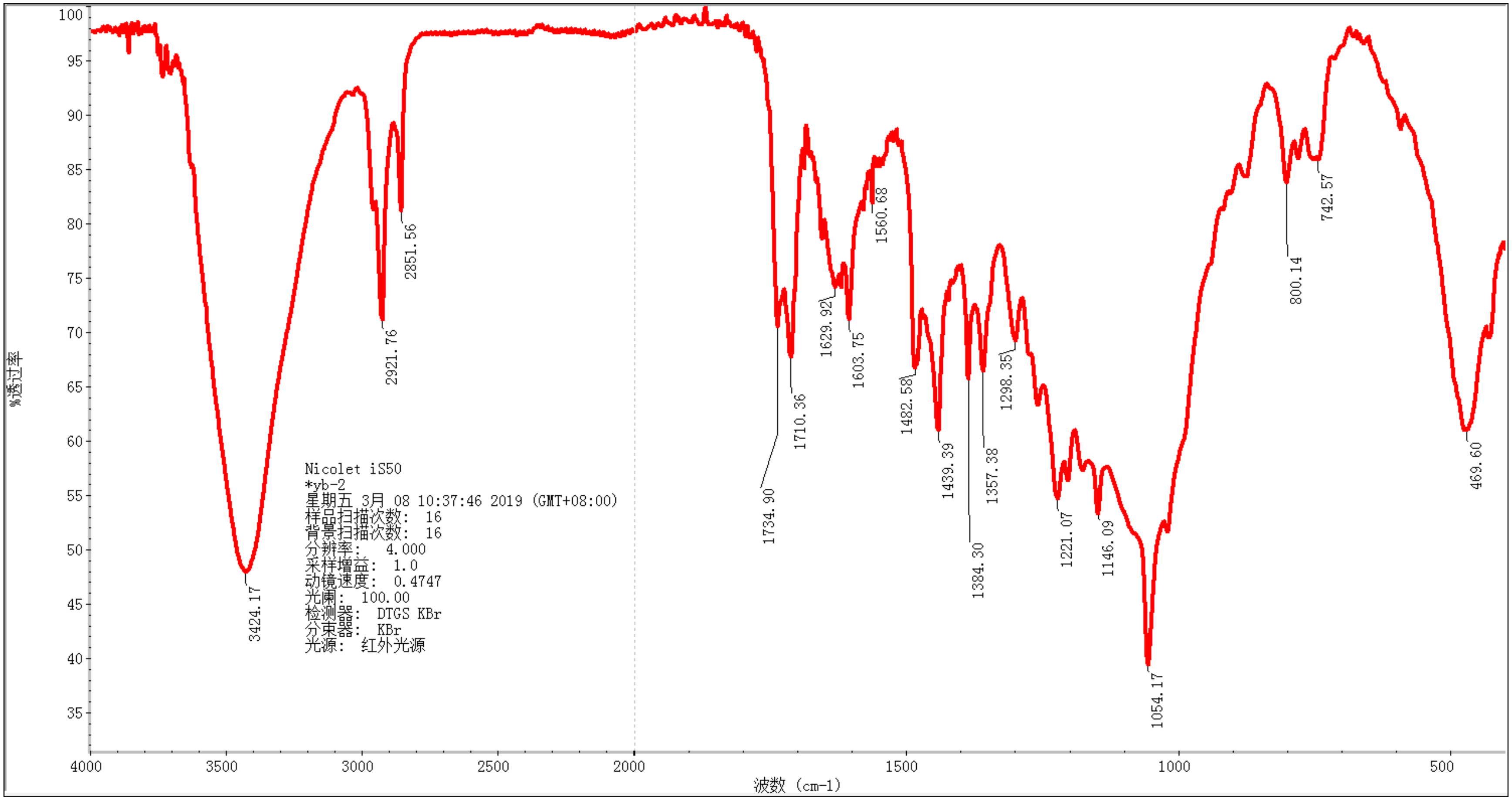

Figure S3. the IR spectrum of IP-2.

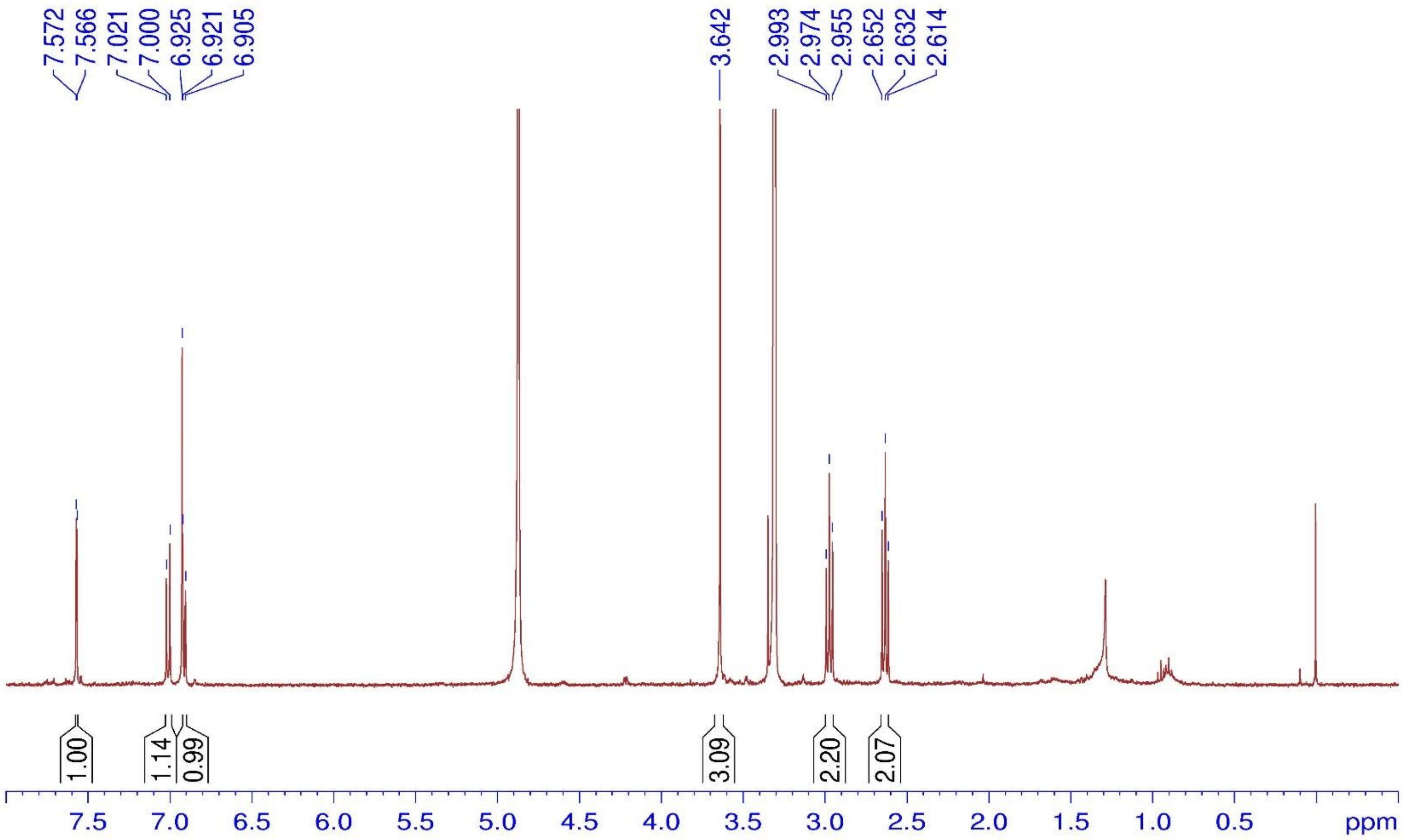

Figure S4. the ^{1}H-NMR spectrum of IP-2 (measured in CD_3OD at 400 MHz).

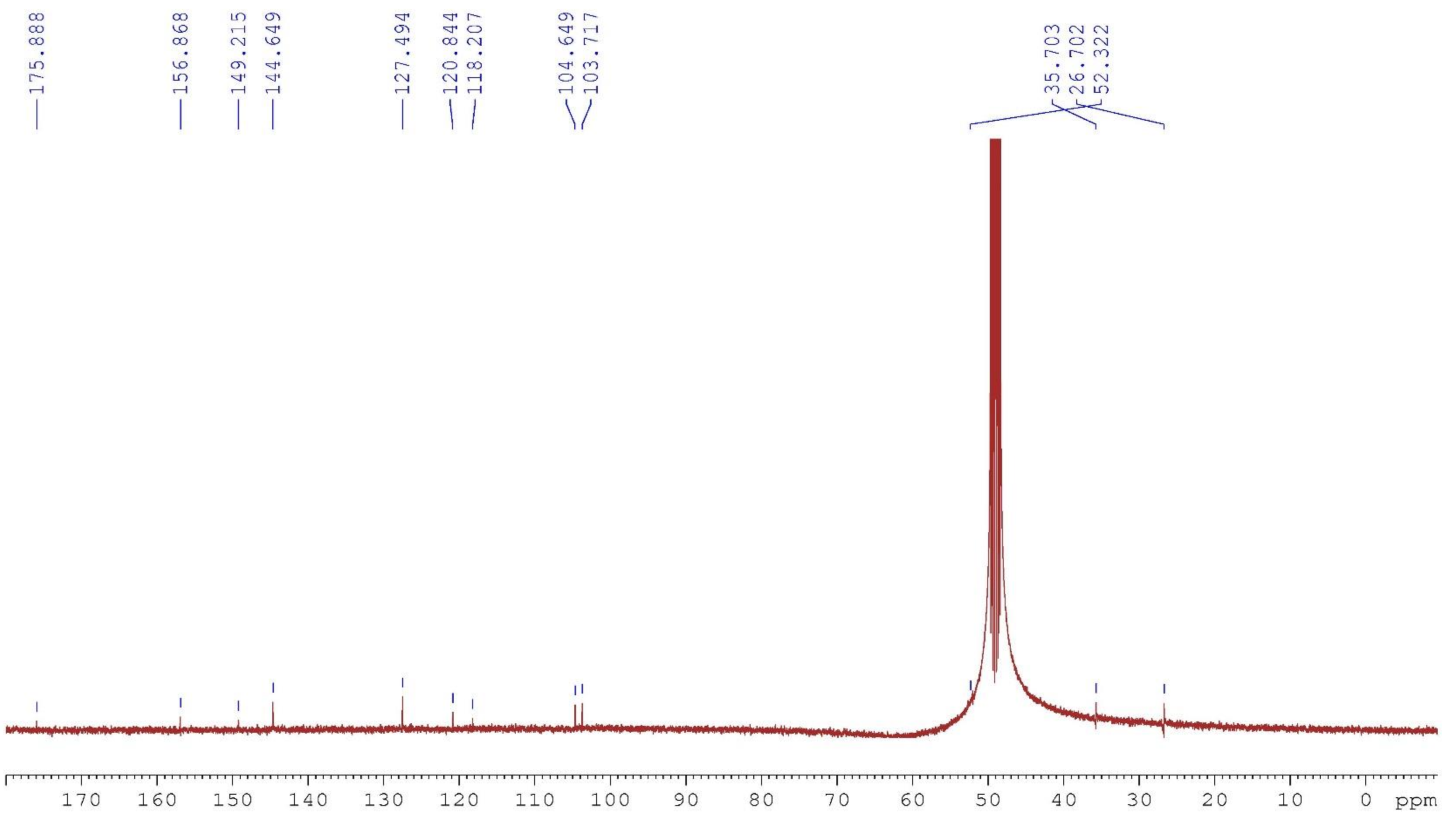

Figure S5. ^{13}C-NMR spectrum of IP-2 (measured in CD_3OD at 400 MHz).

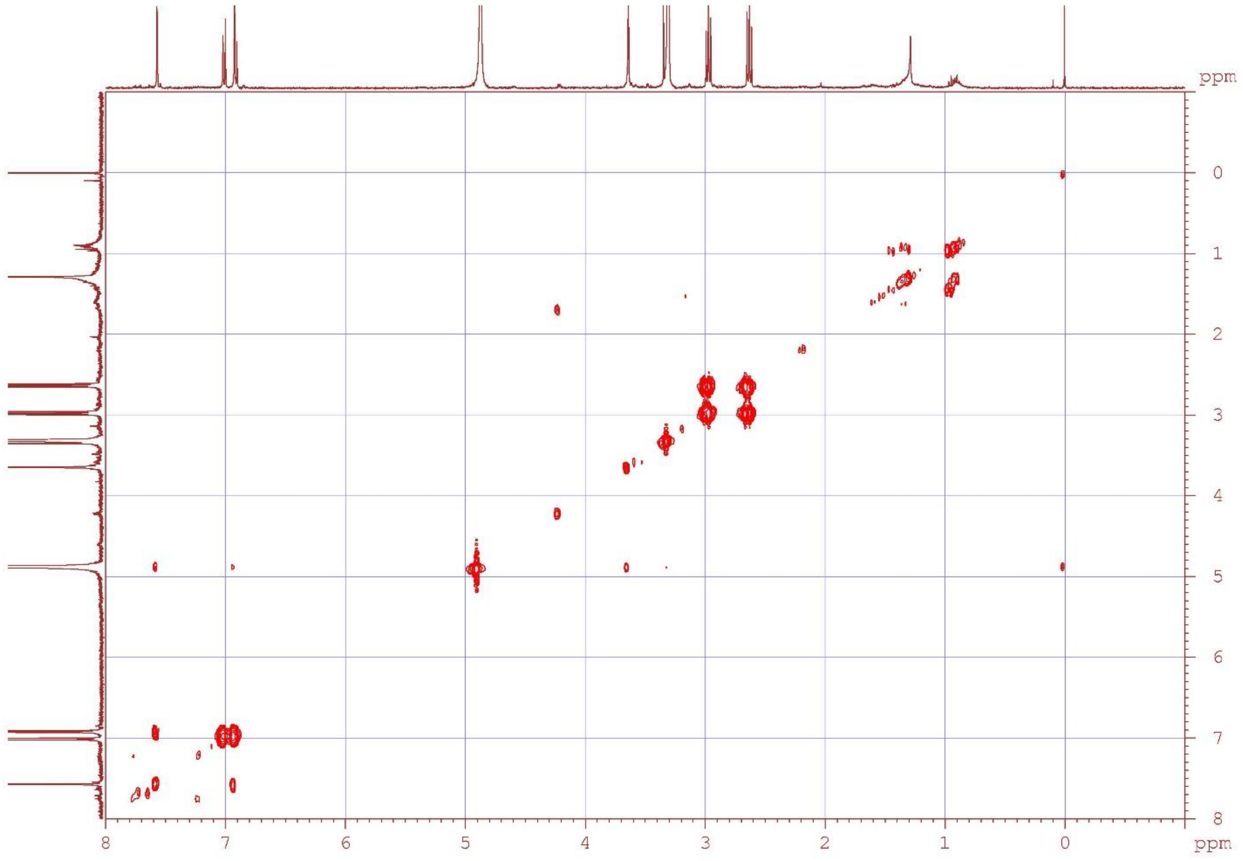

Figure S6. ^{1}H-^{1}H-COSY spectrum of IP-2 (measured in CD_3OD at 400 MHz).

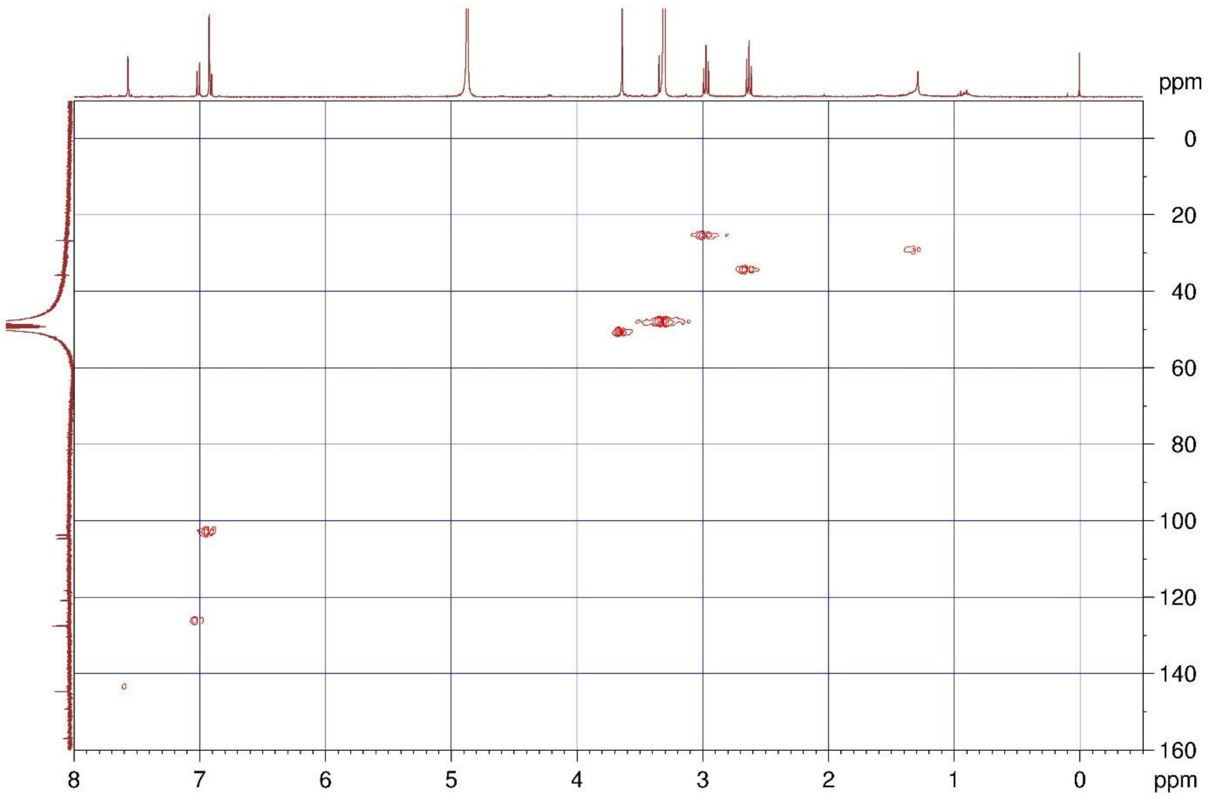

Figure S7. HSQC spectrum of IP-2 (measured in CD_3OD at 400 MHz).

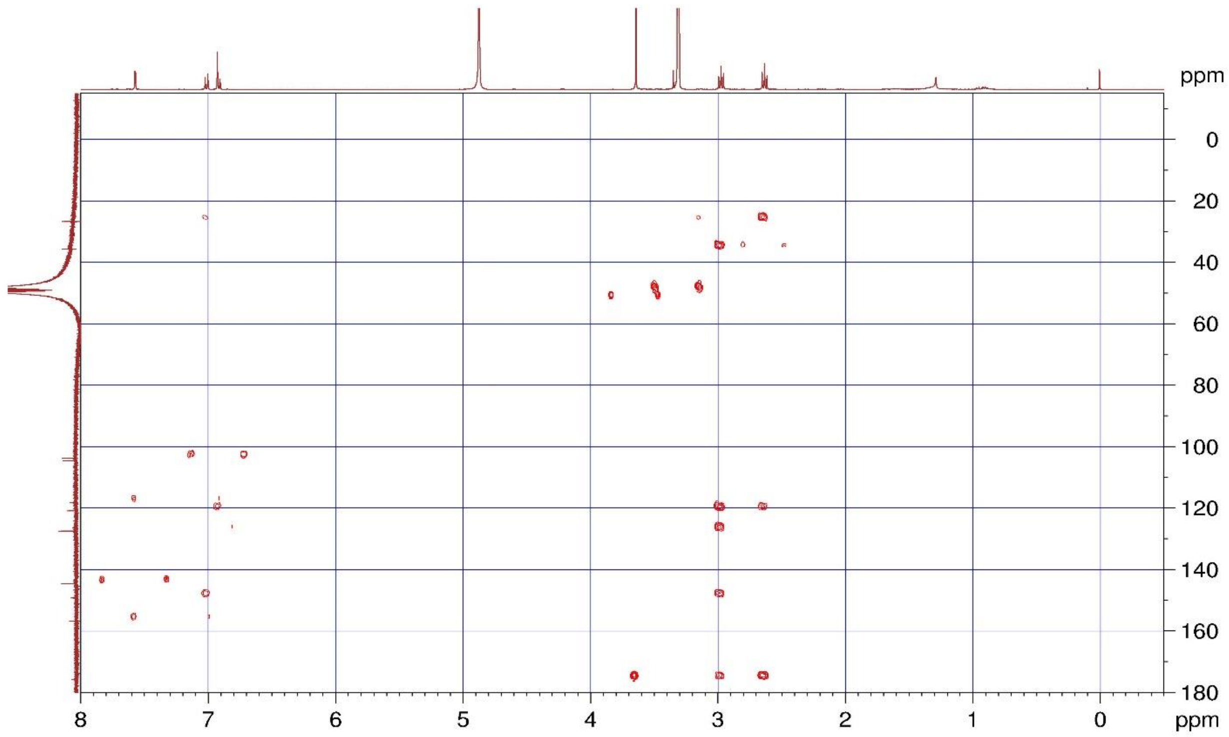

Figure S8. HMBC spectrum of IP-2 (measured in CD_3OD at 400 MHz).

The chemical information of psoralen and isopsoralen.

Psoralen :

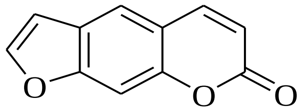

Compound P: white crystals (MeOH); EI-MS *m/z* 186 $[M]^+$; ^{1}H-NMR (400 MHz, $CDCl_3$) δ: 7.80 (1H, d, *J* = 9.6 Hz, H-4), 7.70 (1H, d, *J* = 2.2 Hz, H-2'), 7.68 (1H, s, H-5), 7.46 (1H, s, H-8), 6.84 (1H, d, *J* = 2.2 Hz, H-3'), 6.38 (1H, d, *J* = 9.6 Hz, H-3); ^{13}C NMR (100 MHz, $CDCl_3$) δ: 161.0 (C-2), 114.6 (C-3), 144.0 (C-4), 119.8 (C-5), 124.8 (C-6), 156.3 (C-7), 99.8 (C-8), 152.0 (C-9), 115.4 (C-10), 146.9 (C-2'), 106.3 (C-3'), which is in agreement with previous reports listed in the revised manuscript.

Isopsoralen :

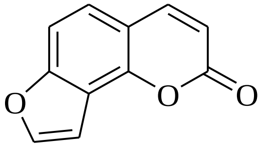

Compound IP: white crystals (MeOH); EI-MS *m/z* 186 $[M]^+$; ^{1}H-NMR (400 MHz, $CDCl_3$) δ: 7.81 (1H, d, *J* = 9.6 Hz, H-4), 7.69 (1H, d, *J* = 2.0 Hz, H-2'), 7.43 (1H, d, *J* = 8.6 Hz, H-5), 7.37 (1H, d, *J* = 8.6 Hz, H-6), 7.12 (1H, d, *J* = 2.0 Hz, H-3'), 6.39

(1H, d, *J* = 9.6 Hz, H-3); ^{13}C NMR (100 MHz, $CDCl_3$) δ: 160.8 (C-2), 114.0 (C-3), 144.5 (C-4), 123.8 (C-5), 108.7 (C-6), 157.3 (C-7), 116.8 (C-8), 148.4 (C-9), 113.4 (C-10), 145.8 (C-2'), 104.0 (C-3'), which is in agreement with previous reports listed in the revised manuscript.

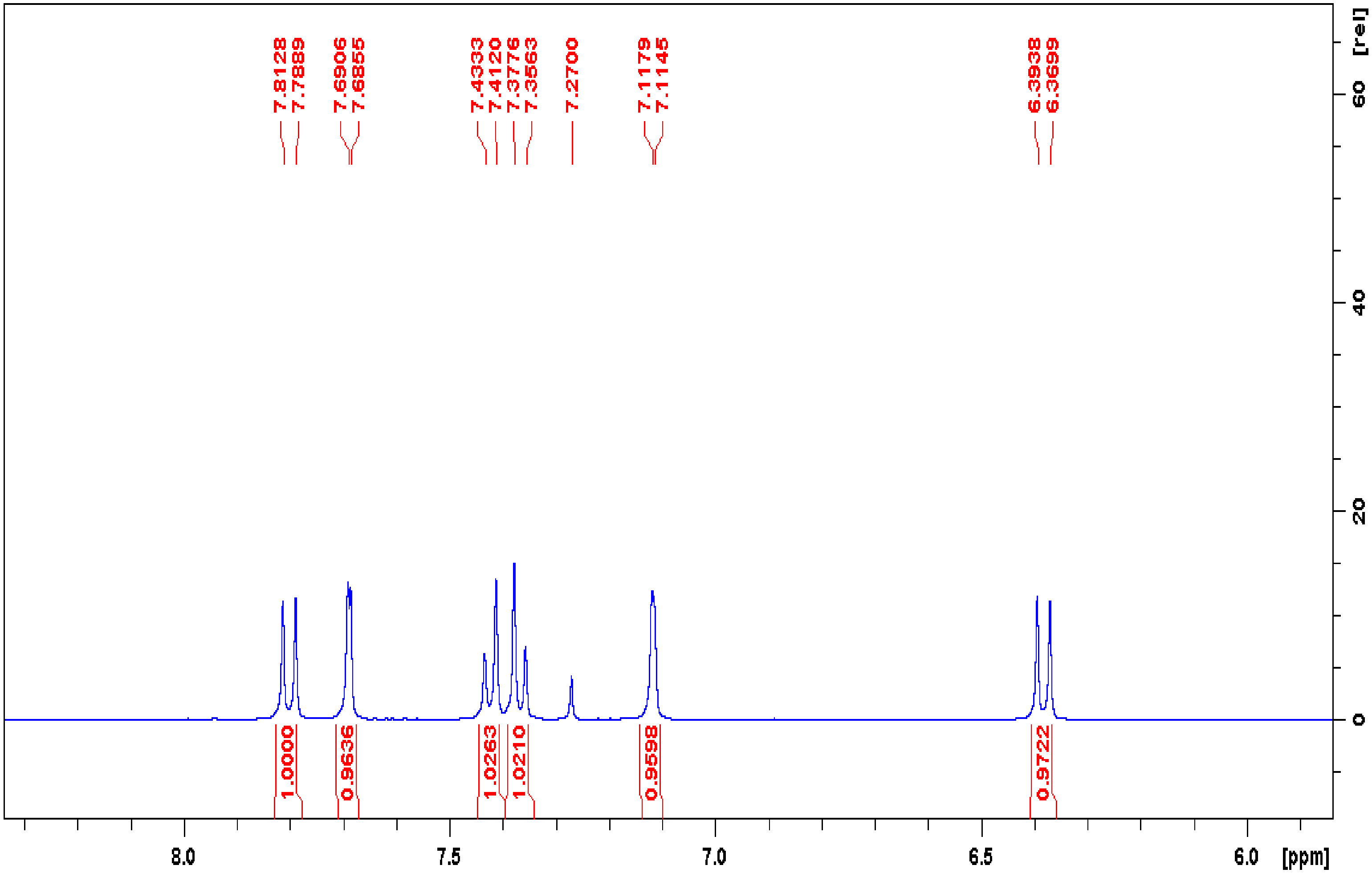

Figure S9. ^{1}HNMR spectrum of P in $CDCl_3$.

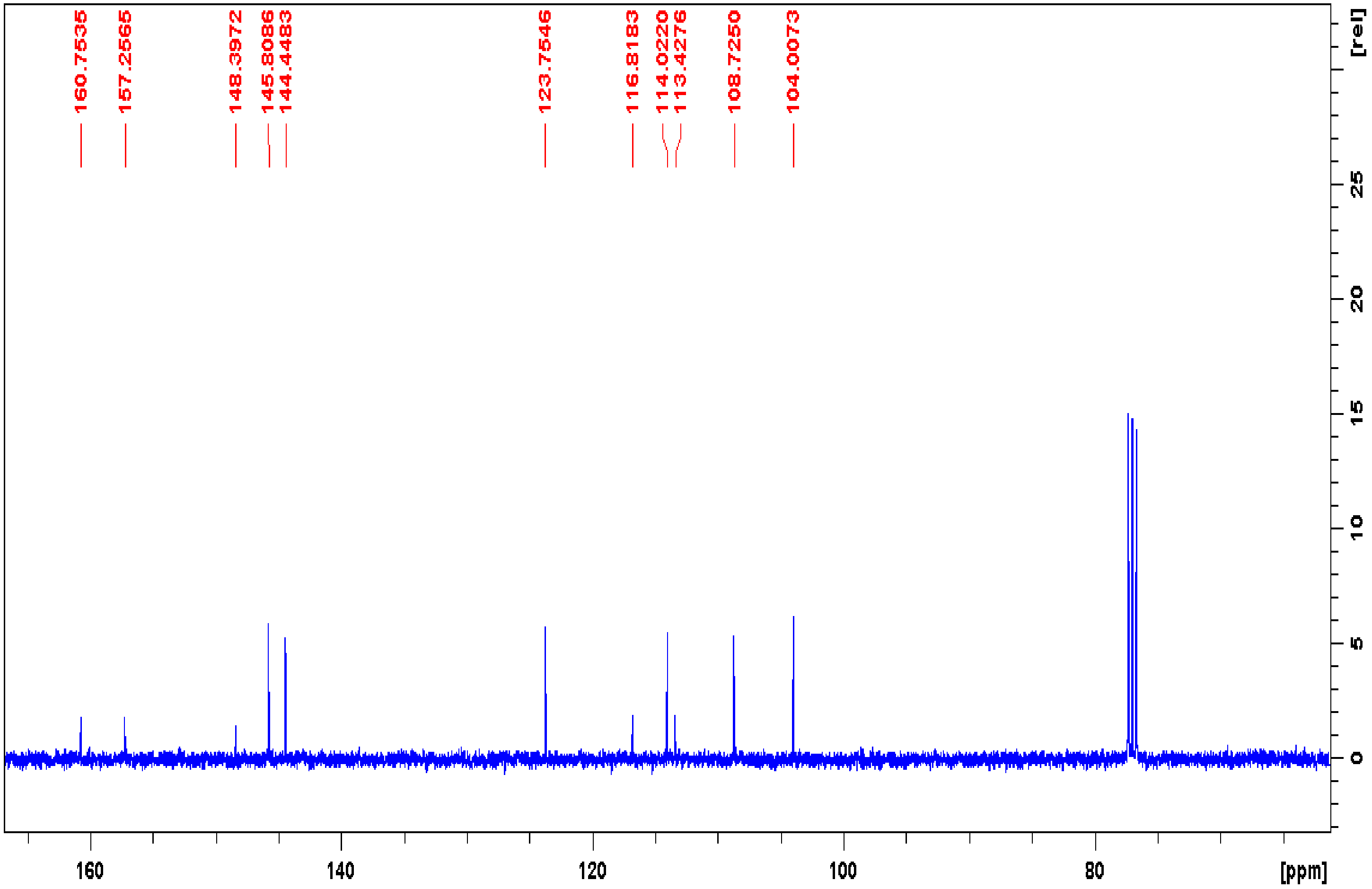

Figure S9. ^{13}CNMR spectrum of P in $CDCl_3$.

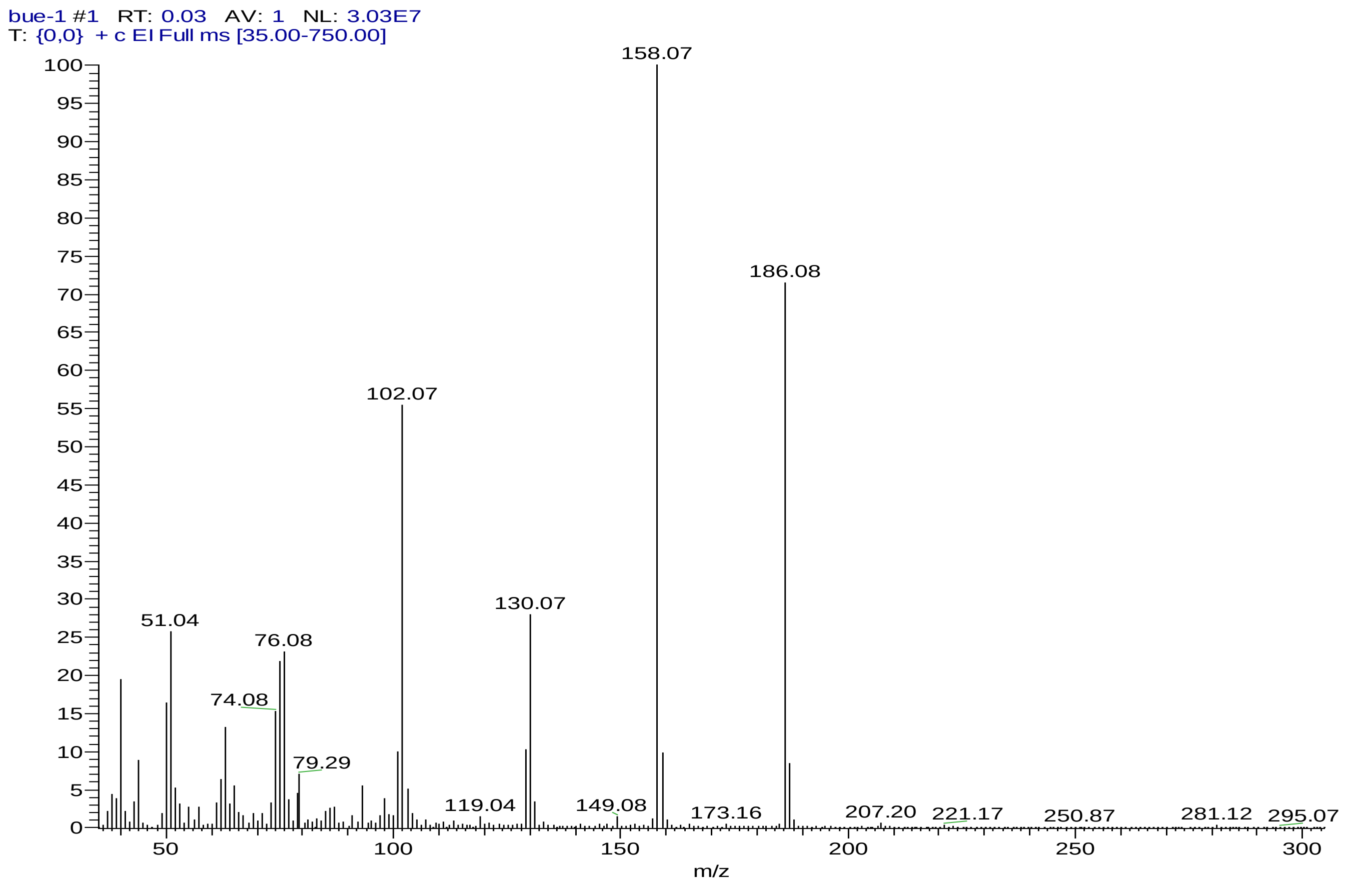

Figure S11. EI-MS spectrum of P.

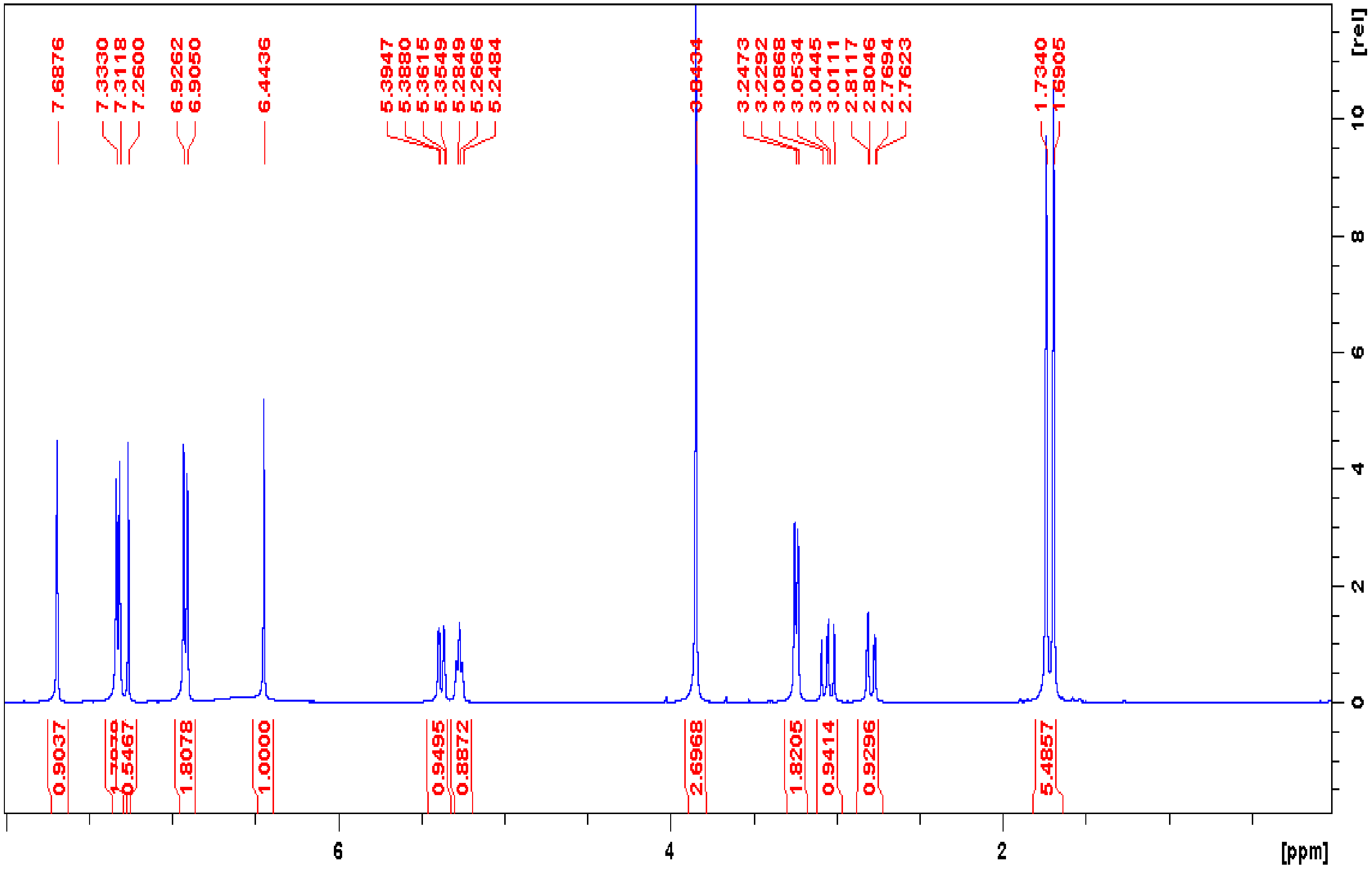

Figure S12. ^{1}HNMR spectrum of IP in $CDCl_3$.

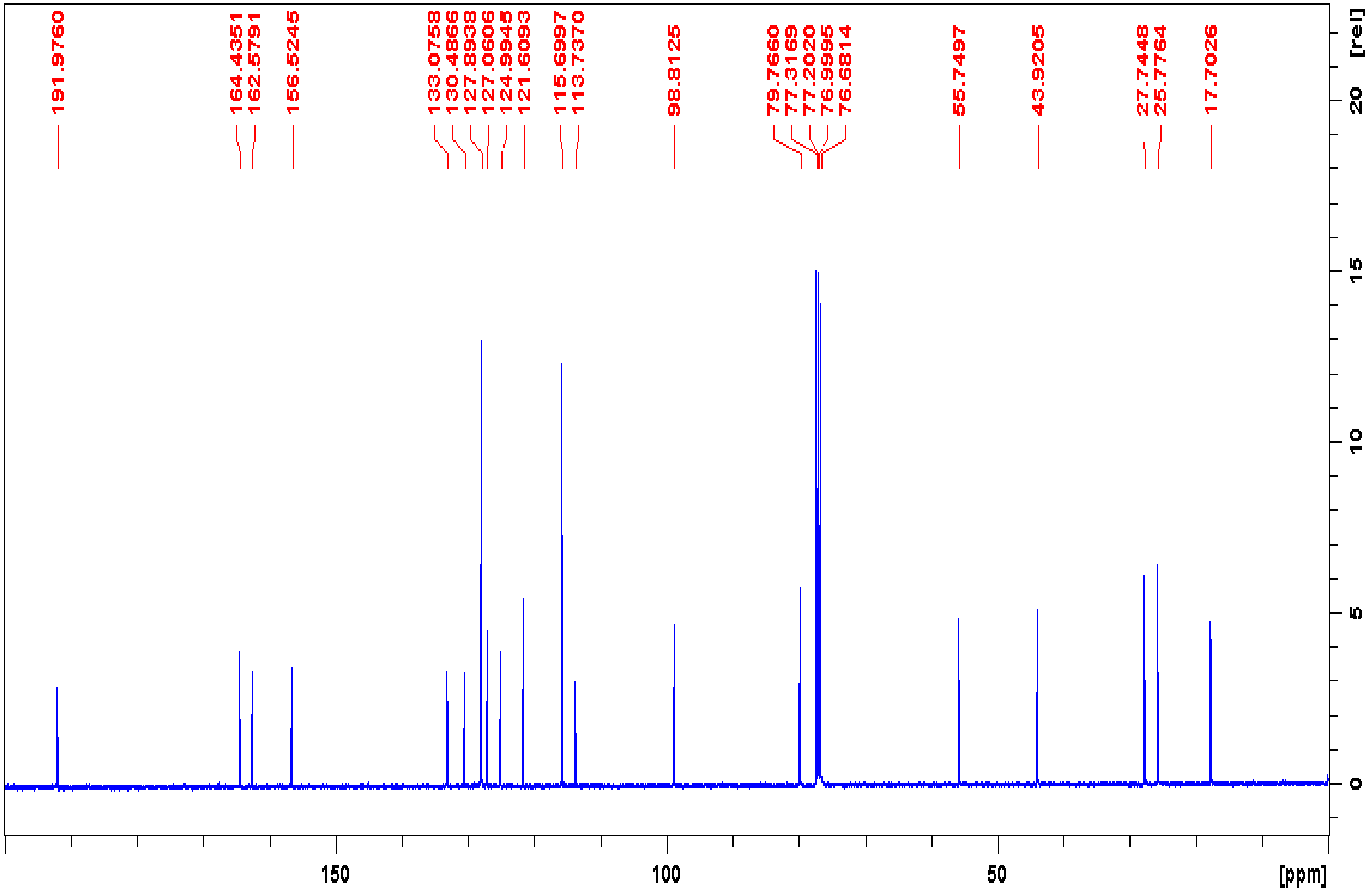

Figure S13. ^{13}CNMR spectrum of IP in $CDCl_3$.

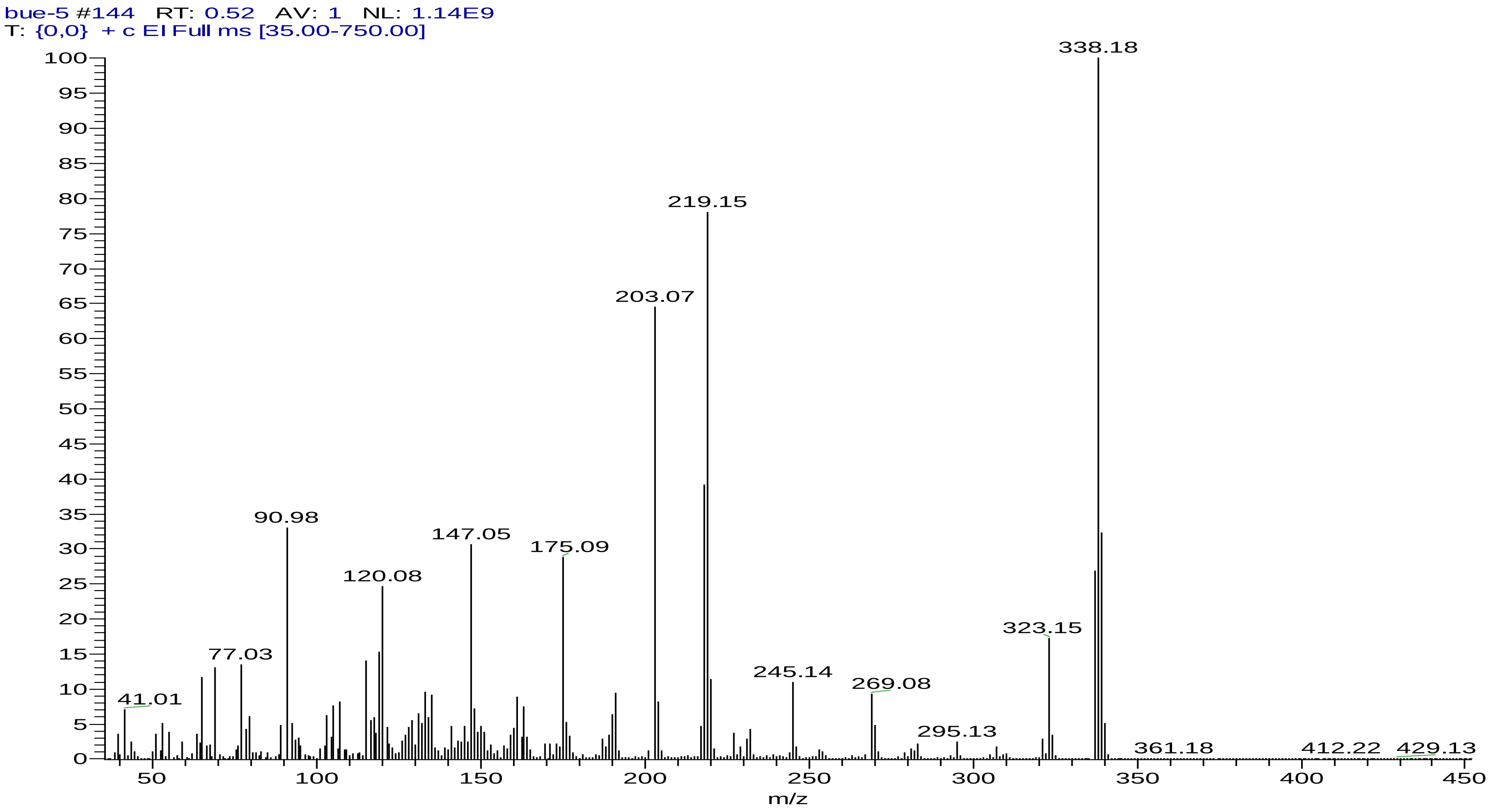

Figure S14. EI-MS spectrum of IP.
